# *BLADE-ON-PETIOLE* genes temporally and developmentally regulate the sheath to blade ratio of rice leaves

**DOI:** 10.1038/s41467-019-08479-5

**Published:** 2019-02-06

**Authors:** Taiyo Toriba, Hiroki Tokunaga, Toshihide Shiga, Fanyu Nie, Satoshi Naramoto, Eriko Honda, Keisuke Tanaka, Teruaki Taji, Jun-Ichi Itoh, Junko Kyozuka

**Affiliations:** 1 Tohoku University, Graduate School of Life Sciences, Sendai, 980-8577 Japan; 2 RIKEN, Center for Sustainable Resource Science, Yokohama, 230-0045 Japan; 3 The University of Tokyo, Graduate School of Agricultural and Life Sciences, Tokyo, 113-8657 Japan; 4 NODAI Genome Research Center, Tokyo University of Agriculture, Tokyo, 156-8502 Japan; 5 Department of Bioscience, Tokyo University of Agriculture, Tokyo, 156-8502 Japan

## Abstract

Axis formation is a fundamental issue in developmental biology. Axis formation and patterning in plant leaves is crucial for morphology and crop productivity. Here, we reveal the basis of proximal-distal patterning in rice leaves, which consist of a proximal sheath, a distal blade, and boundary organs formed between these two regions. Analysis of the three rice homologs of the Arabidopsis *BLADE-ON-PETIOLE1* (*BOP1*) gene indicates that OsBOPs activate proximal sheath differentiation and suppress distal blade differentiation. Temporal expression changes of *OsBOPs* are responsible for the developmental changes in the sheath:blade ratio. We further identify that the change in the sheath:blade ratio during the juvenile phase is controlled by the miR156/SPL pathway, which modifies the level and pattern of expression of *OsBOPs*. *OsBOPs* are also essential for differentiation of the boundary organs. We propose that *OsBOPs*, the main regulators of proximal-distal patterning, control temporal changes in the sheath:blade ratio of rice leaves.

## Abstract (web summary)

Despite the importance of proximal-distal patterning of leaves in cereal productivity, the underlying molecular mechanisms are poorly understood. Here, the authors find that the ratio of sheath to blade in rice leaf shifts depends on the expression levels of *BLADE-ON-PETIOLE* genes.

## Introduction

During leaf differentiation, three main axes, adaxial-abaxial, medial-lateral, and proximal-distal, are established. Among the three axes, the former two are the best understood at the molecular level in Arabidopsis, Antirrhinum and maize^1,2^. At initiation of a leaf primordium, a hypothetical signal from the shoot apical meristem (SAM) triggers the specification of the polarity of the adaxial–abaxial axis. Subsequently, mutual antagonistic interactions between factors determining adaxial and abaxial properties establish the polarity and define the boundary between them. The juxtaposition of the adaxial–abaxial cell types promotes the outgrowth of the lamina along the adaxial and abaxial boundary, giving rise to the formation of the medial-lateral axis. In contrast, the mechanisms controlling proximal-distal axis patterning are less well understood.

Leaves of grass species consist of distinct domains along the proximal-distal axis. The leaf sheath and the leaf blade develop at the proximal and the distal domain, respectively^3^. The ligule and the auricle develop at the junction between the sheath and the blade and are known as boundary organs. There are two major features in proximal-distal patterning in grass leaves, namely, differentiation of distinct regions along the axis and determination of sheath:blade ratio. Regarding the first feature, genes responsible for boundary organ differentiation have been well studied^4–7^. Interestingly, although the boundary organs are formed between sheath and blade, the sheath and blade differentiate even in *liguleless* (*lg*) mutants in which both ligule and auricle are lost^8–10^. This indicates that boundary organ formation and determination of the sheath:blade ratio are independently controlled aspects of proximal-distal patterning. The second feature, determination of sheath:blade ratio, which is defined by positioning of the boundary organs, is only poorly understood. Analysis of a series of gain-of-function mutants of *KNOTTED-LIKE homeobox (KNOX)* genes in maize provided a conceptual framework of sheath and blade differentiation^11–16^. A “knotted” phenotype caused by misexpression of *KNOX* is interpreted as ectopic ligule and auricle formation resulting from re-establishment of the blade/sheath boundary in the blade^17,18^. Therefore, it is assumed that KNOX specifies proximal cues. How KNOX1 is involved in proximal-distal patterning in normal leaf development, however, remains unknown.

The sheath and the blade in grass species play distinct roles; the sheath strengthens the culm and the blade is the major area of photosynthesis. Therefore, the sheath:blade ratio, which is critical for efficient growth, changes as the plant develops, to optimize growth^19^. In rice, the sheath:blade ratio is high at the beginning of the juvenile vegetative phase, gradually decreasing towards the adult vegetative phase (Supplementary Fig. 1). Changes were observed in several traits in the vegetative phase, such as leaf and stem morphology, branching patterns, growth rate and the plastochron interval. This phenomenon, called heteroblasty, occurs in many plant species. Although the patterns of these changes are species-specific, the mechanism controlling the shift from the juvenile to the adult vegetative phase is strikingly conserved. In this mechanism, microRNA156 (miR156) promotes the juvenile vegetative phase^20^^,^^21,22^. miR156 acts by repressing the expression of *SQUAMOSA PROMOTER-BINDING PROTEIN-LIKE* (*SPL*), encoding plant-specific transcription factors. Because the level of miR156 is crucial for the timing of the vegetative shift, it is finely tuned by several factors. In rice, it has been reported that *PETER PAN SYNDROME1 (PPS1)* works upstream of miR156 and regulates the juvenile-adult phase change^23^^,^^24^. In addition, trans-acting siRNA-mediated regulation of *ETTIN*/*AUXIN RESPONSE FACTOR3* (*ARF3*) and *ARF4* is also involved in the coordination of genetic networks to finetune the change in phase^25^. Despite progress in understanding the molecular basis for the shift in the vegetative growth phases, little is known about the link between phase change and the sheath:blade ratio.

The *BLADE-ON-PETIOLE1* (*BOP1*) gene was first identified as a suppressor of lamina differentiation on the petiole in Arabidopsis^26^^,^^27^. Later, the functions of *BOP1*, as well as a closely related homologous gene, *BOP2*, were revealed in floral patterning, abscission zone formation and bract suppression^28^^,^^29^^,^^30^. *BOP* genes are involved in the control of leaf development as well as other variable developmental processes in other species, including nodule formation in legumes^31^^,^^32^^,^^33^, control of axillary bud growth in barley^34^ and maize^35^ and inflorescence development in barley^36^ and tomato^37^. Despite the number of papers describing leaf defects in *bop* mutants, the roles of *BOP* genes in the control of leaf development are still ambiguous. In later studies, the Arabidopsis *bop* phenotype is interpreted as the shift of the blade-petiole junction to the basal side, rather than ectopic lamina proliferation along the petiole, as reported in the original study^38^. A stipule and nectary, small organs borne at the base of the petiole, are lost or reduced in *bop* mutants in pea and *Lotus japonicus*, respectively, suggesting that *BOP* genes control differentiation of the basal part of leaves^31,32^. In tomato, *BOP* represses leaflet formation and is involved in the diversity of leaf complexity^39^. Recently it was reported that *BOP* is required for ligule development in barley, indicating that *BOP* is involved in at least one aspect of proximal-distal patterning in grass species^34^^,^^40^. However, whether *BOP* genes play any roles in determination of the leaf sheath:blade ratio, the main feature of proximal-distal patterning in grass leaves, is unknown.

In this study, we show that *OsBOP*s determine the leaf sheath:blade ratio through activation of proximal sheath differentiation and suppression of distal blade differentiation. *OsBOP*s are also essential for ligule and auricle differentiation. Thus, we propose *OsBOP*s as the main regulators of proximal-distal patterning of rice leaves. In addition, we demonstrate that developmental control of the leaf sheath:blade ratio is achieved by regulation of spatial/temporal, as well as quantitative, patterns of expression of *OsBOP*s. Furthermore, we show that expression of *OsBOP*s is under the control of the microRNA156/SPL pathway.

## Results

### OsBOP genes redundantly control leaf sheath development

The *BOP* genes encode proteins containing a BTB/POX VIRUS AND ZINC FINGER (POZ) domain and ankyrin repeats^41^. Among three *BOP* homologs of rice, *OsBOP1* is the closest homolog of two *BOP* genes in *Arabidopsis*, while *OsBOP2* and *OsBOP3* belong to a grass-specific clade (Supplementary Fig. 2)^27–29^. To examine the function of the three *OsBOP* genes, we generated loss-of-function mutants using the CRISPR system (Supplementary Fig. 3)^42^.

**Fig. 1:**
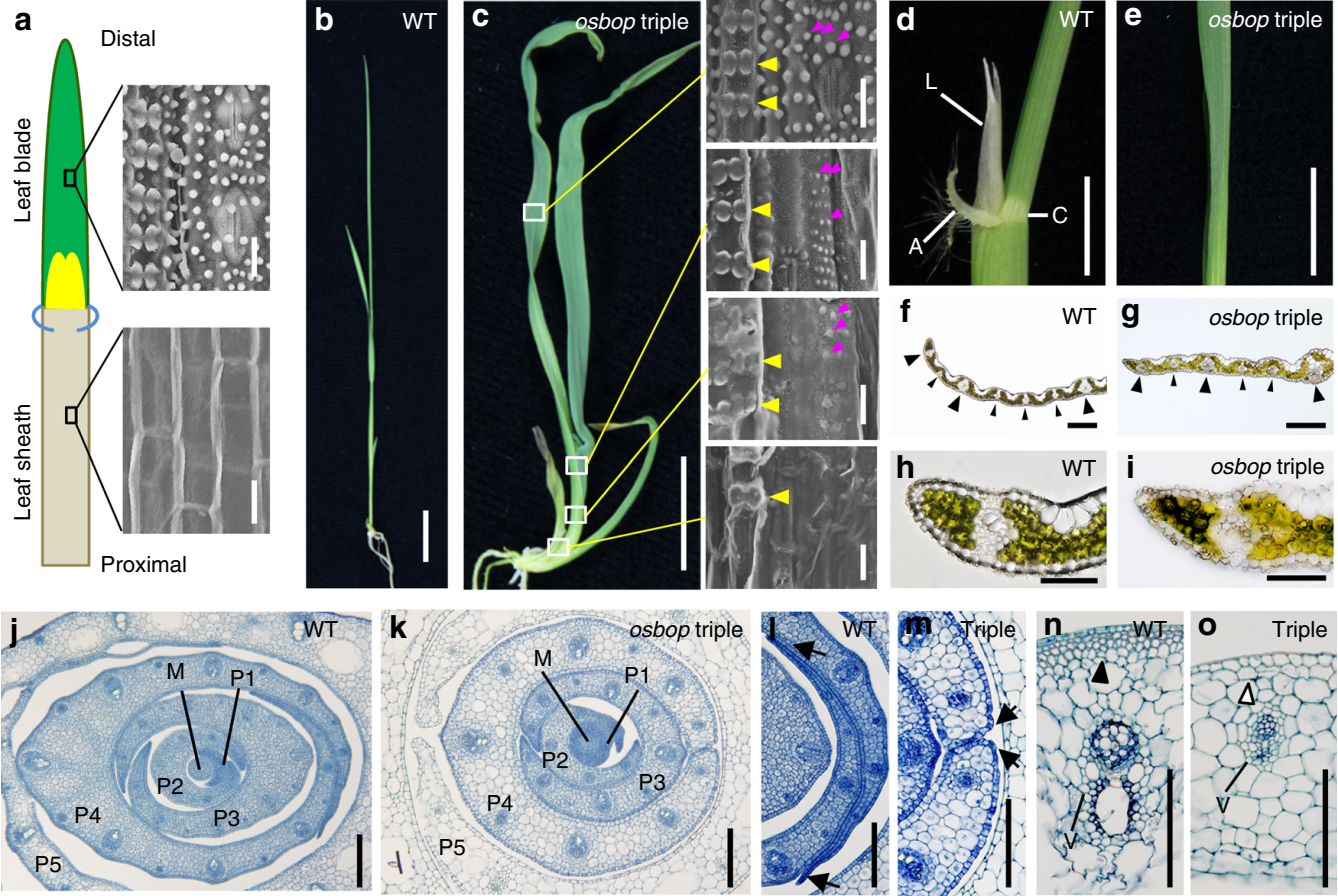
Morphology of *osbop* triple mutants. **a** Structure of a rice leaf. A leaf sheath (gray) and a leaf blade (green) develop at the proximal and the distal position, respectively. Ligules (yellow) and auricles (light blue) form in the junction between the sheath and the blade. SEM views of the adaxial surface of the sheath (lower) and the blade (upper) are shown. **b** A WT seedling. **c** An *osbop1-1 osbop2-1 osbop3-1* triple mutant seedling. SEM views of the adaxial surface of the third leaf in the triple mutant are shown to the right of the panel (Bars idicate 20 μm). White squares indicate the position of the SEM views. Yellow and magenta arrowheads, respectively, indicate dumbbell-shaped cells and protuberances, both of which are absent in the adaxial surface of the leaf sheath, but present in that of the leaf blade. **d** Boundary region between the leaf blade and the leaf sheath in the WT. L, ligule; A, auricle; C, collar. **e** Middle region of a leaf in the triple mutant lacking all organs formed at the leaf blade/sheath boundary in the WT. **f**, **g** Cross-sections of leaf blade in the WT (**f**) and the triple mutant (**g**). Large and small arrowheads indicate large and small vascular bundles, respectively. **h**, **i** Cross-sections of leaf blade margins in the WT (**h**) and the triple mutant (**i**). **j**, **k** Cross-sections of a shoot lower region of the WT (**j**) and the triple mutant (**k**) when the sixth leaf has emerged. M, shoot apical meristem. **l**, **m** Close-up views of the marginal region of the WT (**l**) and the triple mutant (**m**). Arrows indicate leaf margins. **n**, **o** Close-up views of the large vasculature (V) in the lower leaf region of the WT (**n**) and the triple mutant (**o**). An arrowhead in **n** and an open arrowhead in (**o**) indicate abaxial sub-epidermal cells in the WT and the triple mutant, respectively. Bars: 20 μm (**a**), 1 cm (**b**, **c**), 500 μm (**d**, **e**), 200 μm (**f**, **g**), 100 μm (**h**–**o**)

In a wild-type (WT) rice leaf, the leaf sheath and the leaf blade form at the proximal and the distal side, respectively^43^. The two parts can be clearly distinguished by their surface structures on the adaxial side, bumpy on the leaf blade and smooth on the leaf sheath. The ligules and the auricle develop at the junction between the sheath and the blade (Fig. 1a). The gross morphology of the three single mutants was normal, while varied defects, such as reduction in plant height and abnormal development of the ligule and the auricle, were observed in the double mutants (Supplementary Figs. 4, 5). Defects were further amplified in triple mutants. We analyzed three independent alleles of each locus and found that all exhibited the same defects, indicating that the defects were caused by the mutations in the *OsBOP* genes (Supplementary Fig. 3). From these observations, we concluded that the three *OsBOP* genes control leaf development in rice redundantly. Three mutants, one in each locus (*osbop1-1*, *osbop2-1,* and *osbop3-1)*, were chosen for further analysis.

The leaves of the triple mutants were extremely short, dark and curled (Fig. 1b, c). Because the ligules and the auricles were completely lost in the triple mutant (Fig. 1d, e), we observed the adaxial surface structure from top to bottom to determine the identity along the proximal-distal axis in the leaves. At the distal part of the leaf, the adaxial surface showed typical blade characteristics (dumbbell-shaped cells and protuberances, Fig. 1c). An arrangement of vascular bundles (Fig. 1f, g) and a marginal structure (Fig. 1h, i) also indicated blade characteristics. The blade characteristics gradually decreased in severity and a mixture of both blade and sheath characteristics was observed towards the basal part of the leaf, however, dumbbell-shaped cells, one characteristic of the blade, were detected at the bottom of the leaf (Fig. 1c). These observations indicated that most regions of the leaves of the triple mutant exhibited blade characteristics with a basipetal gradient in the severity of the phenotype. This suggests that *OsBOP*s exert their functions to promote leaf sheath differentiation acropetally.

During leaf differentiation, longitudinal and lateral expansion of the leaf primordium begins at the plastochrone 1 (P1) stage when the primordium is the youngest. Both lateral margins of the leaf primordium meet at the P2 stage when the primordium is the second youngest, then, further grow laterally to overlap and wrap inner younger leaves from the P3 to P5 stages (Fig. 1j, l). The lateral growth of the margin is a characteristic feature of the leaf sheath. In the triple mutants, the leaf margins met at the P2 to P3 stages, however, they did not show further lateral growth (Fig. 1k, m). In the leaf of the WT plants, the vasculature is located on the abaxial side and is connected to the epidermis by a few layers of sclerenchyma cells (Fig. 1n). Conversely, in the triple mutant, vascular tissues did not develop fully and sclerenchyma cells adjoining vascular bundles did not differentiate in the leaves (Fig. 1o). Despite the many defects observed in the leaf sheath, few visible abnormalities were observed in the distal part of the blade. Based on these observations we concluded that the *OsBOP* genes promote normal development of the leaf sheath and the ligule, and suppress leaf blade development at the distal position. In addition to the leaf defects, inflorescences in the single and double mutants were smaller than in the WT, however, no clear defects in flower patterning were observed.

### Expression pattern of the OsBOPs during leaf development

**Fig. 2:**
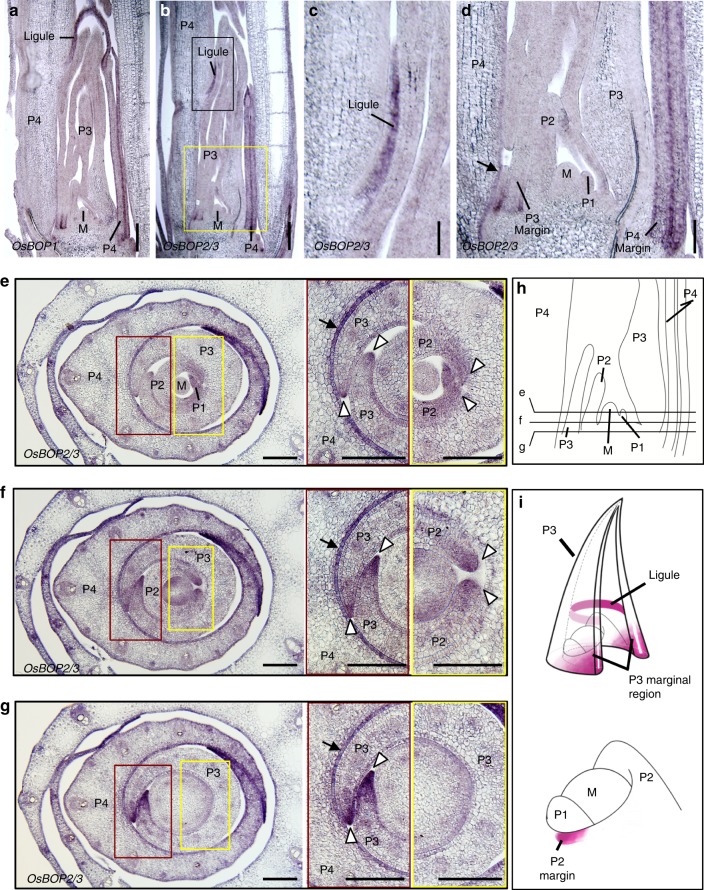
Spatial localization of *OsBOP* transcripts at the sixth leaf stage (**a**–**d**) Localization of *OsBOP1* mRNA (**a**) and *OsBOP2/3* mRNA (**b**–**d**) in the longitudinal section of the region around the shoot tip at the sixth leaf stage. A close-up of the black or yellow square in **b** is shown in **c** and **d**, respectively. **e**–**g** Localization of *OsBOP2/3* mRNA in the transverse sections of the lower part of the shoot at the sixth leaf stage. A close-up of the brown and yellow squares in **e**–**g** are shown to the right of the panels. The position of sections is depicted in **h**. Arrows in **e**–**g** indicate the adaxial epidermis in the P4 leaf. Open arrowheads in **e**–**g** indicate leaf margins. **h** Schematics of the longitudinal section in **d**. **i** Schematic of the in situ signals in P2 and P3 leaves. The P3 leaf surrounding the inner leaf primordia and the shoot apical meristem is shown (top). The marginal regions of the P3 leaf are shown as sectional planes. A close-up of P1 and P2 leaf primordia and the shoot apical meristem (bottom). The BOP signal is depicted in purple. M, shoot apical meristem. Bars: 200 μm (**a**, **b**), 100 μm (**c**, **d**, **e**–**g**)

We next determined the expression pattern of the *OsBOP* genes in the leaf of the adult vegetative stage by in situ hybridization analysis. As it was technically challenging to distinguish between *OsBOP2* and *OsBOP3* due to a high level of similarity in the nucleotide sequences, we detected the transcripts of *OsBOP2* and *OsBOP3* simultaneously. Young plants, at the sixth leaf stage when the leaf sheath:blade ratio is almost at its minimum (Supplementary Fig. 1), were used for the analysis. In both longitudinal and transverse sections, the expression patterns observed between *OsBOP1* and *OsBOP2/3* were predominantly conserved (Fig. 2, Supplementary Fig. 6). We confirmed that the expression levels of *OsBOP1* and *OsBOP2/3* in *osbop1,2,3* triple mutants were undetectable (Supplementary Fig. 6). In longitudinal sections, *OsBOP1* and *OsBOP2/3* expression was first observed in the epidermal cells at the marginal side of the base of the leaf primordia at the P3 stage (Fig. 2a, b, d). *OsBOP1* and *OsBOP2/3* signals were observed at the base of the leaf margin at the P3 stage and these signals extended to the ligule position in P4 leaves (Fig. 2a, b, d). This indicated that the signals extended upwards to the position of the ligule during development from the P3 to the P4 stage (Fig. 2a–c). *OsBOP1* and *OsBOP2/3* signals were detected in the ligule in the stage P4 leaf primordia (Fig. 2a–c).

The expression patterns of *OsBOP1* and *OsBOP2/3* were also examined in serial transverse sections (Fig. 2e–g, Supplementary Fig. 6). In the section at the distal position, which includes the tip of the SAM in the middle, the signal was detected at the margins of the leaves at the P2, P3, and P4 stages and in the epidermal cells in the P4 and P5 leaves (Fig. 2e). In the transverse sections, the signal at the margin of the P3 leaves became stronger and larger in area in the basal sections (Fig. 2e, f–h). The signal was observed throughout the epidermis in leaves at the P4 stage, indicating that the expression started from the marginal region at the base of the leaf primordia and expanded to the central region of the leaf (Fig. 2e–g). Noticeably, the signal was excluded from the SAM (Fig. 2b, d, e).

In summary, *OsBOP1* and *OsBOP2/3* expression started from the base of the lateral margins of the leaf primordium at the P2 stage. The expression in the epidermis then expanded centrally and upwards, consistent with the acropetal function of *OsBOPs* revealed by the mutant phenotypes. In leaves at the P3 and P4 stages, *OsBOP1* and *OsBOP2/3* expression became strong in the leaf sheath margins. *OsBOP1* and *OsBOP2/3* were also expressed in ligule primordia (Fig. 2i).

### The OsBOP genes determine the shape of the first leaf

**Fig. 3:**
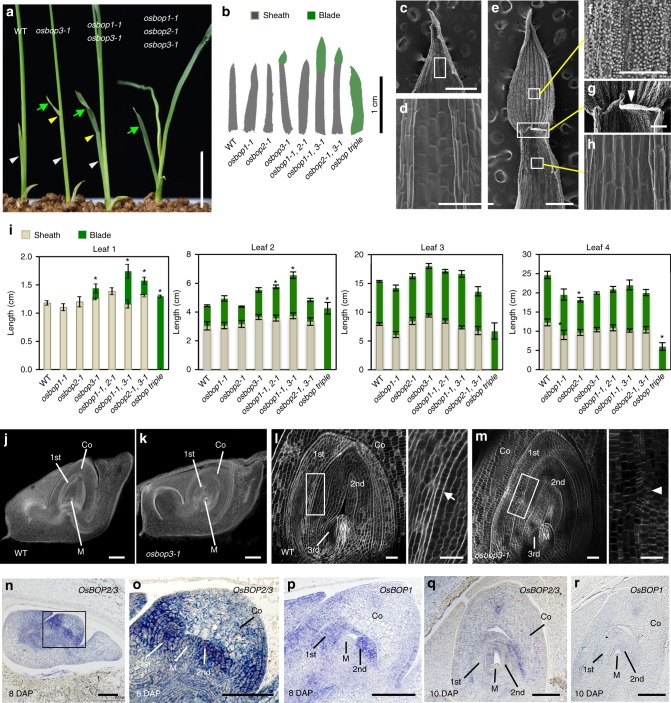
Leaf blade formation in the first leaf of *osbop* mutants. **a** Seedlings of WT, *osbop3-1*, *osbop1-1 osbop3-1* and *osbop1-1 osbop2-1 osbop3-1* mutants. Green arrows, yellow arrowheads and white arrowheads indicate the leaf blade, blade/sheath boundary and leaf sheath, respectively, in the first leaves. **b** The shapes of first leaves in WT and *osbop* mutants. **c**–**h** SEM views of the adaxial side of the first leaf in WT (**c**, **d**) and *osbop3-1* plants (**e**–**h**). A close-up of the white square in **c** is shown in **d**. A close-up of the white squares in **e** is shown in **f**–**h**, respectively. An arrowhead in **g** indicates the ligule. **i** The length of the leaf blade and sheath of the first four leaves in the WT and *osbop* mutants. Error bars indicate standard error (*n* = 5 biologically independent samples for WT, single and double mutants; *n* = 2 biologically independent samples for triple mutant) Asterisks indicate a statistically significant difference compared to WT (Dunnett’s test, *P* < 0.05). **j**–**m** Confocal images of mature embryos in WT (**j**, **l**) and *osbop3-1* plants (**k**, **m**). A close-up of the white square in **l** and **m** is shown to the right of the panels, respectively. A white arrow indicates the epidermal cell layer of the first leaf in WT (**l**). A white arrowhead indicates ligule initiation in the epidermal cell layer of the first leaf in *osbop3-1* (**m**). **n**, **p** Localization of *OsBOP2/3* mRNA in developing embryos eight days (**n**, **o**) and 10 days (**q**) after pollination. A close-up of the black square in **n** is shown in **o**. **o**, **q** Localization of *OsBOP1* mRNA in developing embryos eight days (**p**) and 10 days (**r**) after pollination. CO, coleoptile; M, shoot apical meristem. Bars: 1 cm (**a**), 500 μm (**c**–**e**), 100 μm (**f**–**h**), 20 μm (**j**–**m**), 100 μm (**n**–**r**). Source data for Fig. 3i are provided as a Source Data file

After germination, the first leaf consists of leaf sheath alone (Fig. 3a, b). In the *osbop3-1* mutant, an ectopic leaf blade differentiated on the distal side of the first leaf (Fig. 3a, b). The identity of the ectopic leaf blade was confirmed by its rough surface on the adaxial side (Fig. 3c–h). Differentiation of the boundary organs was also confirmed (Fig. 3g). Although an ectopic leaf blade was formed in *osbop3-1* alone among the three single mutants, the leaf blade area in the first leaf was larger in the double mutant, and the leaf blade occupied the most area in the triple mutant (Fig. 3a, b, i, Supplementary Fig. 7). These data indicate that the first leaf has the potential to develop a leaf blade on the distal side, but this ability is suppressed by the redundant actions of the three *OsBOP* genes.

In rice, differentiation of the first leaf occurs during embryogenesis^43^. Therefore, we further analyzed the *osbop3-1* phenotype at this stage. We followed ligule primordia as an indicator of leaf blade differentiation. The first step of ligule initiation is periclinal divisions at a single cell layer of the adaxial epidermis^44^^,^^45^. A protruded region with an extra cell layer derived from this periclinal division was observed in the first leaf primordia of *osbop3-1*, but not in WT mature seeds and seeds after 12 h imbibition (Fig. 3j–m, Supplementary Fig. 8). Expression of *OsLIGULELESS1*/*OsSPL8*, which is required for ligule formation^46^, supports our hypothesis that this region corresponds to the initiating ligule (Supplementary Fig. 8). Our analysis in mature seeds indicates that the proximal-distal patterning in the first leaf is already started in *osbop3-1* before germination.

We then analyzed the developing embryo eight days after pollination (8 DAP) when the first and second leaf primordia initiate. Consistent with the notion that *OsBOP*s function to suppress leaf blade differentiation in the first leaf, *OsBOP* genes were expressed in the WT embryo at 8 DAP (Fig. 3n–p). The signals of *OsBOP1* and *OsBOP2/3* were high in the entire region of the first and second leaf primordia and the proximal part of the coleoptile at 8 DAP (Fig. 3o, p), while they became weaker and were restricted to the marginal regions of the first leaf at 10 DAP (Fig. 3q, r). These data imply that the expression of *OsBOP*s during embryogenesis is developmentally controlled to evoke temporally high expression when the first and second leaf primordia initiate.

### Expression of OsBOPs is developmentally regulated

**Fig. 4:**
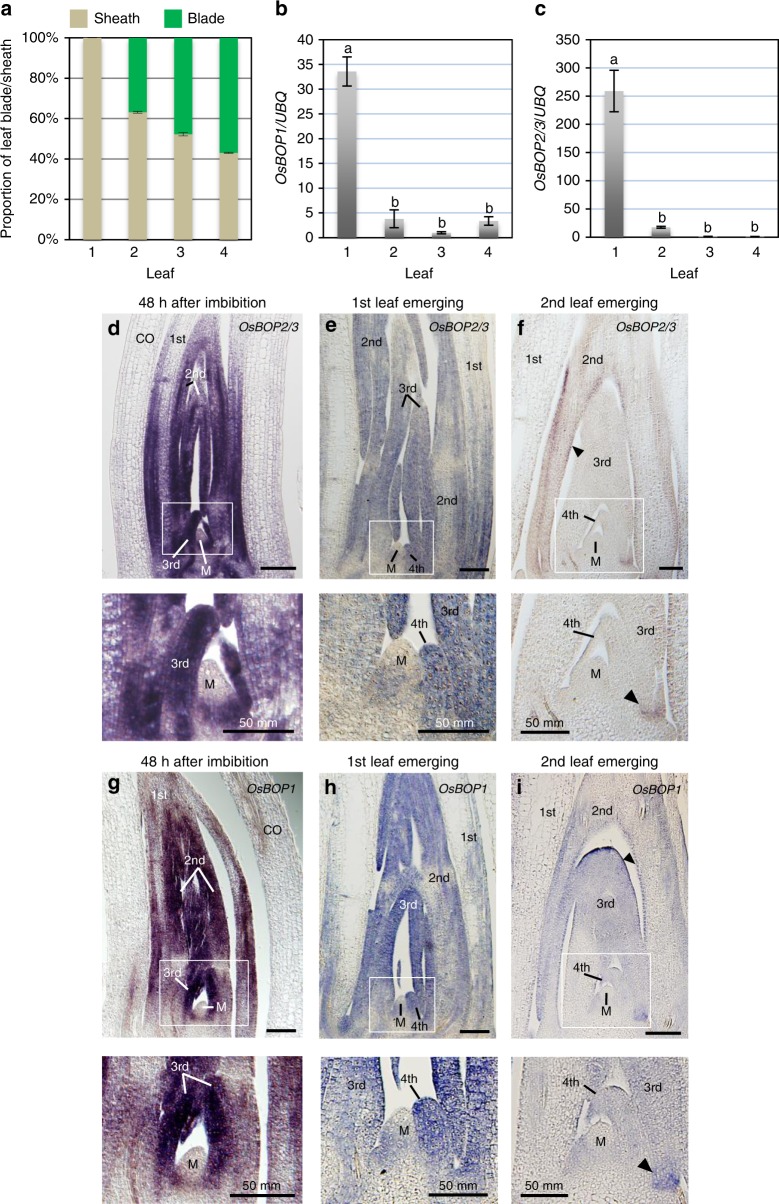
Expression patterns of the *OsBOP* genes in juvenile leaves. **a** The proportion of leaf blade and sheath in the first four leaves of WT. Error bars indicate standard error (*n* = 3 biologically independent samples). **b**, **c** Relative expression levels of *OsBOP1* (**b**) and *OsBOP2/3* (**c**) in the first four leaves. Each leaf was collected just as it was emerging from the coleoptile (the first leaf) or from the sheath of the previous leaf (the second to fourth leaf). The relative expression value of the third leaf is shown as 1.0. Error bars indicate standard error (*n* = 3 biologically independent samples). The first leaf has significantly higher expression levels of *OsBOP1* and *OsBOP2/3* than the others (Tukey-Kramer test, *P* < 0.05). The significant difference is indicated by different signs on the graph. **d**–**i** Localization of *OsBOP2/3* mRNA (**d**–**f**) and *OsBOP1* mRNA (**g**–**i**) in germinating shoots 48 h after imbibition (**d**, **g**), at the emergence of the first leaf (**e**, **h**) and at the emergence of the second leaf (**f**, **i**). A close-up view of the white squares in **d**–**i** is shown immediately below the panel. Arrowheads indicate the epidermis of the second leaf primordia. Arrowheads indicate lower part of the third leaf. CO, coleoptile; M, shoot apical meristem. Bars: 100 μm (**d**–**i**). Source data of Fig. 4a–c are provided as a Source Data file

The results described above suggested that the developmentally regulated change of the sheath:blade ratio could be due to a temporal change in OsBOP activity. We hypothesized that a basal level of OsBOP activity is required to promote normal proximal sheath development and a further increase in OsBOP activity is needed to inhibit distal blade differentiation. To test this hypothesis, we first analyzed the temporal changes in expression levels of the *OsBOP*s. Consistent with our prediction, *OsBOP1* and *OsBOP2/3* expression was highest in the first leaf primordia in which blade differentiation is strongly inhibited, followed by a sudden decrease in the second leaf primordium (Fig. 4a–c). Expression was maintained at a low level in the second to fourth leaves.

To further clarify differences in the expression pattern of *OsBOP*s in the second to fourth leaves, we performed in situ hybridization analysis in WT seedlings at three different stages: 48 h after seed imbibition, emergence of first leaf and emergence of second leaf. In the seeds after 48 h imbibition, when the primordia of the second and third leaves are at the P2 and P1 stages, respectively, a strong signal from both *OsBOP1* and *OsBOP2*/*3* was detected in the entire region of the second and third leaf primordia (Fig. 4d, g). It is noteworthy that, despite strong expression in the leaf primordia, *OsBOP1* and *OsBOP2/3* expression was excluded from the SAM. In the seedling after the emergence of the first leaf, when the fourth leaf primordium is at the P1 stage, the *OsBOPs* signal was present in the entire region of the fourth leaf primordium but with a much-reduced intensity (Fig. 4e, h). The *OsBOP*s signal became much weaker and was restricted to a specific region in the seedling at the emergence of the second leaf (Fig. 4f, i). Overall, the onset of expression of the *OsBOP*s was observed in the whole area of the primordia at the P1 stage in second, third and fourth leaves, while the intensity of expression was weaker in the later-arising leaf. Furthermore, down-regulation occurred earlier in later-arising leaves. These observations indicated that the expression of *OsBOP1* and *OsBOP2/3* is strictly regulated spatially, temporally and quantitatively. *OsBOP*s are expressed more strongly and for longer periods in the earlier-arising leaf primordia in which the sheath:blade ratio is higher, than in the later-arising leaf primordia in which the sheath:blade ratio is lower.

### The OsBOP genes regulate spikelet organ development

**Fig. 5:**
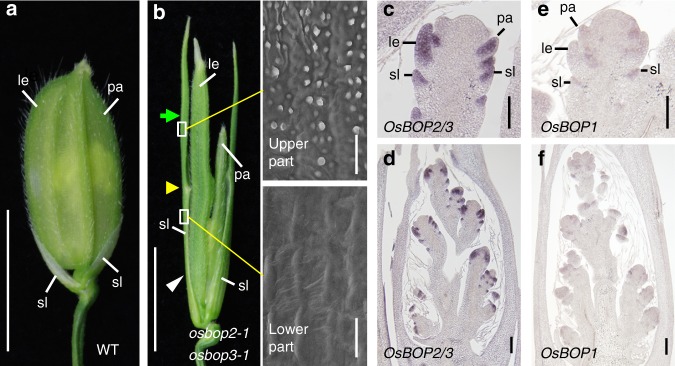
Morphology of the spikelet in *osbop2-1 osbop3-1*. **a** A WT spikelet. Arrowheads indicate sterile lemmas. **b** An *osbop2-1 osbop3-1* spikelet. A green arrow, yellow arrowhead and white arrowhead indicate the upper part, upper/lower boundary and lower part, respectively, in a sterile lemma. SEM view of an adaxial surface in the upper and lower part of the mutant sterile lemma is shown to the right of the panel (Bars indicate 20 μm). **c**, **d** Localization of *OsBOP2/3* mRNA in developing spikelets. **e**, **f** Localization of *OsBOP1* mRNA in developing spikelets. le, lemma; pa, palea; sl, sterile lemma. Bars: 5 mm (**a**, **b**), 50 μm (**c**, **e**), 100 μm (**d**, **f**)

In rice, after the transition to the reproductive phase, severely suppressed consecutive leaves form in the inflorescence, followed by a few sets of glumes. In these modified leaves, the blade is vestigial (Fig. 5a). We asked if *OsBOP*s are involved in the control of leaf sheath and blade proportion in these reproductive-phase leaves. While *osbop1-1* and *osbop2-1* did not show any obvious phenotype in the spikelet organs, sterile lemmas were longer in *osbop3-1* single and *osbop1-1 osbop3-1* double mutants (Supplementary Fig. 9). The *osbop2-1 osbop3-1* plants showed more severe defects in which sterile lemmas elongated to form two distinctive parts along the proximal-distal axis, with boundary organs forming between them (Fig. 5b). The upper part of the sterile lemma exhibited the rough surface characteristics of the leaf blade (Fig. 5b). in situ hybridization experiments revealed that *OsBOP2/3* is strongly expressed in the entire region of developing spikelet organs (Fig. 5c, d). This is consistent with our hypothesis that strong and constitutive expression of *OsBOP*s leads to suppression of the leaf blade. In contrast to *OsBOP2/3*, *OsBOP1* expression was not detected in the spikelet (Fig. 5e, f), which is consistent with the lack of obvious phenotype in the spikelet organs of the *osbop1* mutants (Supplementary Fig. 9).

### OsBOPs are sufficient to promote leaf sheath development

**Fig. 6:**
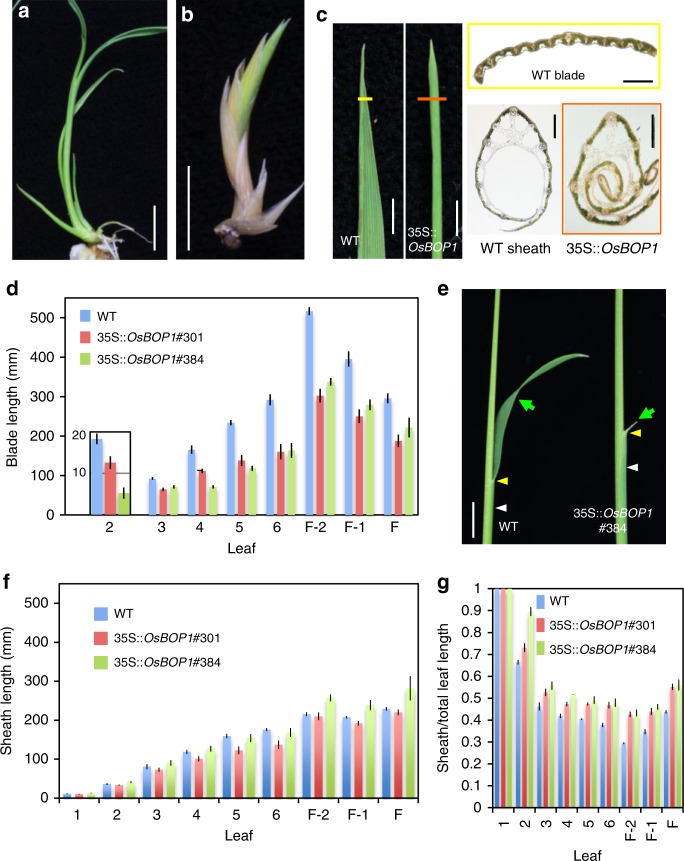
Effects of constitutive overexpression of *OsBOP1* (**a**, **b**) non-transformed (**a**) and 35s::*OsBOP1* (**b**) plantlets. **c** The tip of leaves in the WT and in 35s::*OsBOP1* with severe effects. Cross-sections at regions indicated with yellow and orange bars are shown in yellow and organ squares right to the panels, respectively (Bars indicate 200 μm). **d** The average length of leaf blade. ‘F’ indicates the flag leaf, which is the last leaf. The blade length in the second leaf is shown at one-tenth the scale in the *y*-axis. The blade length in both 35s::*OsBOP1* lines shown in **d** is shorter than that in the WT (Dunnett’s test, *P* < 0.05). **e** View of WT and 35s::*OsBOP1* #384 seedlings. Green arrows, yellow arrowheads and white arrowheads indicate the leaf blade, blade/sheath boundary and leaf sheath, respectively, in the second leaf. **f**, **g** The average length of leaf sheath (**f**) and the ratio of sheath/total leaf length (**g**). The ratio of sheath/total leaf length in both 35s::*OsBOP1* lines is higher than that of the WT with the exception of the first leaf in #384 and the first to third leaves in #301 (Dunnett’s test, *P* < 0.05). The length of the leaf sheath in both 35s::*OsBOP1* lines is not statistically different from that of the WT with the exception of the fifth and sixth leaves in #301 and of the F-2 and F-1 leaves in #384 (Dunnett’s test, *P* < 0.05). **h**–**j** The leaf blades in 35 s::*OsBOP1* # 384. Error bars indicate standard error (*n* = 6 biologically independent samples for WT; *n* = 5 biologically independent samples for #301 and #384,). Bars: 1 cm (**a**, **b**, **d**), 3 mm (**c**, **e**). Source data of Fig. 6d, f, and g are provided as a Source Data file

The clear correlation between the level of *OsBOPs* expression and the sheath:blade ratio prompted us to test whether ubiquitous and high expression of the *OsBOPs* is sufficient for leaf sheath differentiation. As the amino acid sequences in OsBOP2 and OsBOP3 are very similar, we expressed *OsBOP1* or *OsBOP2* under the control of the constitutive cauliflower mosaic virus (CaMV) 35 s promoter (Supplementary Fig. 10). Most of the transformed calli turned brown and died without regenerating plantlets (see Supplementary Fig. 10a, Class III). The level of *OsBOP* expression was high in the calli that failed to regenerate plantlets. Among the regenerated plants, severely affected plantlets produced several short, sheath-like leaves and then died (Fig. 6a–c, Supplementary Fig. 10). All leaves in the *OsBOP1*ox plants displayed a characteristic leaf sheath, even near the distal tip (Fig. 6c). This indicates that enhanced expression of the *OsBOP* genes is sufficient to inhibit differentiation of the leaf blade. Regenerated plants with less severe effects, in which a much lower level of *OsBOP* expression was observed, grew to maturity and set seeds.

The effects of *OsBOP1* misexpression on the development of the leaf sheath and blade were further examined in T1 progeny of two weak lines of 35s::*OsBOP1* (#301 and #384). Leaves in the T1 plants consisted of the leaf blade, the boundary organs and the leaf sheath, while the blade was significantly shorter than that in WT (Dunnett’s test, *P* < 0.05, Fig. 6d, e), indicating that enhancement of *OsBOP1* activity leads to suppression of leaf blade development. On the other hand, the sheath length was largely unaffected (Fig. 6f). As a result, the ratio of leaf sheath:total leaf length became higher in the T1 plants (Fig. 6g).

### OsBOPs are under the control of the microRNA156/SPL pathway

**Fig. 7:**
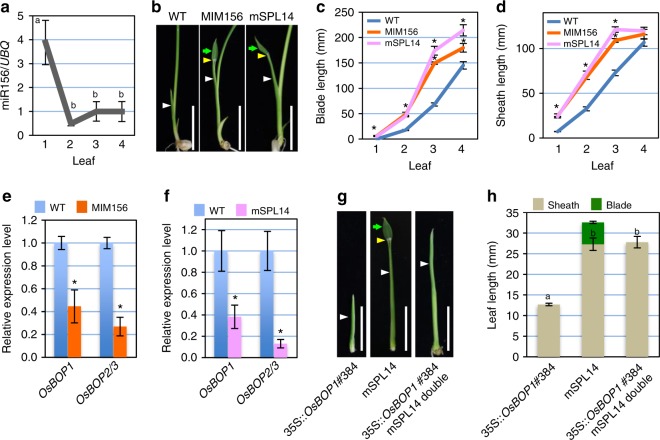
miR156/SPL pathway regulates juvenile leaf traits in rice. **a** Relative expression level of miR156 in the first to fourth leaves in the WT (*n* = 3 biologically independent samples). The expression level of miR156 in the first leaf is higher than that in the other leaves (Tukey-Kramer test, *P* < 0.05). **b** The first leaf phenotype in the WT, in MIM156 and in mSPL14 lines. **c**, **d** The average length of leaf blade (**c**) and sheath (**d**) in WT, MIM156 and mSPL14 lines (*n* = 5 biologically independent samples) Asterisks indicate a statistically significant difference compared to WT (Dunnett’s test, *P* < 0.05). **e**, **f** *OsBOP1* and *OsBOP2/3* expression levels in the first leaf in WT and MIM156 lines (**e**) and in WT and mSPL14 lines (**f**). Each first leaf was collected when it was emerging from the coleoptile (*n* = 3 biologically independent samples). Asterisks indicate a statistically significant difference compared to WT (Student’s *t*-test, *P* < 0.05). **g** First leaf morphology in 35s::*OsBOP1* #384 (left), mSPL14 (middle) and an F1 plant carrying both 35s::*OsBOP1* and mSPL14 constructs (right). **h** The average length of leaf blade and sheath in the first leaves in F2 siblings (*n* = 6 biologically independent samples for 35s::*OsBOP1*; *n* = 3 biologically independent samples for mSPL14; *n* = 12 biologically independent samples for 35s::*OsBOP1* and mSPL14 double line) (Tukey-Kramer test, *P* < 0.05). The relative expression values in the third leaf (**a**) and in the WT (**e**, **f**) are shown as 1.0. Green arrows, yellow arrowheads and white arrowheads indicate the leaf blade, blade/sheath boundary and leaf sheath, respectively, in the first leaves (**b**, **g**). Error bars indicate standard error (**a**, **c**–**f**, **h**). Bars: 1 cm (**b**, **g**). Source data of Fig. 7a, c–f and h are provided as a Source Data file

It has been well documented that juvenile traits are promoted by miR156^21^^,^^47^. In rice, a gradual decrease in miR156 abundance from the second leaf to the fifth leaf was reported^23^^,^^48^, however, the behavior of miR156 in the first leaf is unknown. We determined the level of mature miR156 accumulation in the first to fourth leaves of WT seedlings. qPCR analysis showed the highest level of miR156 accumulation occurred in the first leaf, followed by a rapid decrease in the second leaf. The miR156 level was maintained at a low level in the subsequent leaves (Fig. 7a).

miR156 suppresses expression of the *SPL* family genes^49–51^. To examine the effects of miR156 on the control of leaf development, we generated miR156 target mimicry lines (MM156) in which the target sequence of miR156 is expressed under the CaMV 35s promoter so that the effects of miR156 are reduced through squelching of the target site^51^. Among 19 *OsSPL* genes annotated in the rice genome, 11 contain the miR156 target sequence^49^. We validated increases in expression of most of the *OsSPL* genes with the miR156 target sequence (Supplementary Fig. 11a, b). In contrast, expression of non-target *OsSPL* genes was not significantly changed (Student’s *t*-test, *P* < 0.05). We also generated a miR156-resistant *OsSPL14* (mSPL14) line, in which the miR156 target sequence was changed to a non-target sequence^50^. *OsSPL14* expression was specifically up-regulated in the mSPL14 line (Supplementary Fig. 11c, d)^52^.

An ectopic leaf blade differentiated on the first leaf in both the MM156 and the mSPL14 lines (Fig. 7b). This indicates that the miR156/SPL pathway is involved in the control of the morphology of the first leaf and an increased expression of *OsSPL14* is sufficient to induce the leaf blade on the first leaf. In addition, other traits related to the juvenile/adult phase transition in rice, including the length of the leaf blade and the leaf sheath, the relative midrib length, length/width ratio of a leaf blade and the plastochron, the leaf emergence interval, were altered in both MIM156 and mSPL14 lines (Fig. 7c, d and Supplementary Figs 11, 12) as reported previously^51^.

We then analyzed the genetic relationship between miR156 and *OsBOPs* in the control of the sheath/blade ratio. First, we confirmed that the levels of *OsBOP1* and *OsBOP2/3* were reduced in the first leaf of MIM156 and mSPL14 lines by qPCR, which suggested that *OsBOPs* work downstream of miR156*/OsSPL*s (Fig. 7e, f). In situ hybridization analysis showed that the levels of expression of both *OsBOP1* and *OsBOP2/3* were significantly lower in the mSPL14 line compared to those in the WT, although a clear alteration in the spatial distribution of the expression was not observed (Fig. 4d–i, Supplementary Fig. 13). To further test the genetic interaction, we crossed the 35s::*OsBOP1*#384 line with the mSPL14 line. The ectopic leaf blade differentiation phenotype in the first leaf of the mSPL14 line was abolished in the F1 plant carrying both 35s::*OsBOP1*#384 and mSPL14 (Fig. 7g). This indicates that an increase in *OsBOP1* expression is sufficient to inhibit the effect of increased *OsSPL14*, which enhances leaf blade development. On the other hand, the length of the leaf sheath in the mSPL14 line was not affected by the introduction of 35s::*OsBOP1*#384 (Fig. 7h). This implies that the effects of *OsSPL14* in inducing leaf blade differentiation are independent from its effects in increasing the sheath length.

**Fig. 8:**
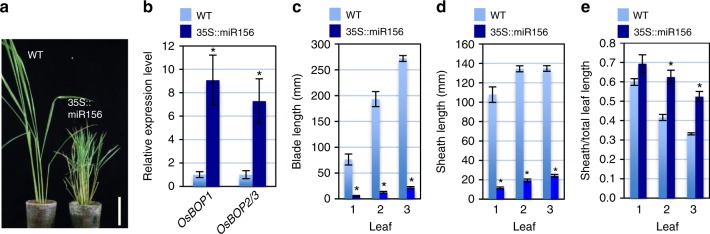
Overexpression of miR156 affects the sheath:blade ratio. **a** The shoot morphology of the WT and 35s::miR156 line. Bar: 10 cm. **b** Relative expression levels of *OsBOP1* and *OsBOP2/3* in third leaves in the WT and 35s::miR156 line (*n* = 3 biologically independent samples). The relative expression values in the WT is shown as 1.0. Both *OsBOP1* and *OsBOP2/3* expression levels in the 35s::miR156 line are statistically different from those in the WT (Student’s *t*-test, *P* < 0.05). **c**–**e** The leaf blade length (**c**), the leaf sheath length (**d**) and the ratio of sheath/total leaf length (**e**) in the first three leaves in axillary shoots (*n* = 9 biologically independent samples for WT; *n* = 8 biologically independent samples for 35s::miR156 line). The length of leaf blade and sheath in first to third leaves in 35s::miR156 is statistically different from those in the WT (Student’s *t*-test, *P* < 0.05). The ratio of sheath/total leaf length in second and third leaves in 35 s::miR156 lin is statistically different from those in the WT (Student’s *t*-test, *P* < 0.05). Error bars indicate standard error (**b**–**e**). Asterisks indicate a statistically significant difference compared to WT (**b**–**e**). Source data of Fig. 8b–e are provided as a Source Data file

We also analyzed the effects of miR156-overexpression on leaf development (Fig. 8a–e). Because the 35s::miR156 plants remained at the vegetative phase and never produced seeds, so we analyzed the phenotypes in the tillers of T0 generation plants. We confirmed that *OsBOP* mRNA accumulation was highly upregulated in leaves of 35s::miR156 plants (Fig. 8b). In addition, although the length of both leaf sheath and leaf blade was reduced in the 35s::miR156 plants, the proportion of the leaf sheath was increased (Fig. 8c–e). These data support our hypothesis that *OsBOP*s work downstream of miR156/SPL to modulate proximal/distal patterning of leaves.

## Discussion

We revealed that *OsBOP*s regulate the sheath:blade ratio in two ways. The first is by the promotion of proximal sheath development, which occurs throughout the plant’s lifetime. The second is by the inhibition of distal blade differentiation, which occurs during the juvenile vegetative phase and the reproductive phase, when the leaf sheath is predominant. Based on these data, we propose that *OsBOP*s work as the acropetal sheath factor and the balance between *OsBOP*s and the predicted, as yet unknown, blade factor, which works in a basipetal manner, determines the position of the border between the sheath and blade along the proximal-distal axis. Elucidation of the mechanism(s) by which *OsBOPs* work as the proximal sheath factor, the actual nature of the blade factor, and their interactions, will be the next challenge in further understanding the proximal-distal patterning in leaves.

We also revealed the tight correlation between the expression pattern of *OsBOP*s and the sheath:blade ratio, suggesting that a change in *OsBOP*s expression is responsible for the developmental phase-dependent shift of the sheath:blade ratio. Our detailed analysis of the change in *OsBOP*s expression associated with the progression of development during the early juvenile phase, when the second to fourth leaf primordia initiate, revealed that *OsBOP*s expression starts in the entire area of the primordia at the P1 stage in all three leaves. The level of expression, as well as its duration, however, was not the same in the three leaves so that the expression was high and maintained for a longer period in earlier-arising leaves. This multifaced fine-tuning of *OsBOP*s expression is likely to be the key that ensures the gradual change in the leaf sheath/blade ratio throughout development. Although we showed that expression of *OsBOP*s is under the control of miR156/SPL, no miR156 target sequence exists in the *OsBOP* genes and *OsBOP*s are not bound by OsSPL14^53^. Recently, it was reported that *BOP* is directly activated by *teosinte branched (tb1)* to regulate axillary bud growth in maize^35^. In rice, *FC1*, the ortholog of maize *tb1*, is bound by OsSPL14^53,54^, indicating that *FC1* may be the link between *OsSPL14* and *OsBOP*s. Because *OsSPL14* and *FC1* are expressed in the whole area of the leaf primordia^52,55^, there may be additional regulators that restrict *OsBOP*s expression to the proximal epidermis during the adult reproductive phase.

The developmental phase-dependent change of morphological traits is called heteroblasty. The sheath:blade ratio is one such heteroblastic trait. Besides the sheath:blade ratio, heteroblasty is observed in other traits in rice leaves. We showed that all these traits are under the control of the miR156/SPL pathway, whereas only the sheath/blade ratio is affected by the change in *OsBOPs* expression (Supplementary Fig. 14). This implies that *OsBOP*s were recruited downstream of the miR156/SPL module to modify the leaf sheath:blade ratio in a developmental phase-dependent manner.

The boundary organs, the ligule and auricle, are formed between the leaf sheath and the leaf blade^43^. They are absent in leaves that consist of only one sheath and blade, such as the first leaf and spikelet organs in WT, *osbop* triple mutant and severe *OsBOP* overexpression plants. In contrast, we showed that the boundary organs were restored in the first leaf in *osbop3* mutants associated with differentiation of the leaf blade. These data indicate that differentiation of both leaf sheath and leaf blade is a prerequisite for boundary organ formation. However, differentiation of the sheath and the blade does not depend on the presence of boundary organs because, even in mutants that completely lack the ligule and auricle in maize and rice, the sheath and the blade differentiate normally^8–10^^,^^46^. Despite the leaf sheath and the leaf blade differentiating normally at the expected position along the distal/proximal axis, the partial loss of the ligule formation phenotype in *osbop1* single mutants is consistent with this data. These defects also indicate that *OsBOP*s are involved in boundary organ formation directly through promotion of ligule differentiation and indirectly through determination of the sheath:blade ratio. The fact that *OsBOP*s expression initiates at two discontinuous domains in a leaf primordium, namely the ligule primordium and the base of the leaf primordium, supports the hypothesis that the two roles of *OsBOP*s in proximal-distal patterning in leaf development are independent.

In Arabidopsis, the phenotype of the *bop1* and *bop2* loss-of-function mutants was first interpreted to be lateral outgrowth of the leaf blade along the petiole caused by the ectopic occurrence of meristematic cells along the petiole^26,29^. Thus, *BOP* function in leaf development was explained as a suppression of meristematic activity of cells. Subsequently, however, Ichihashi et al.^38^ discovered that a proliferative zone is established at the junction between the leaf blade and leaf petiole shortly after leaf primordia initiation in Arabidopsis. Their finding, that *BOP*s are necessary for the correct positioning of the proliferative zone, indicated that *BOP* genes are required for the patterning of the leaf petiole and blade. Our study has revealed that *OsBOP* genes control the sheath:blade ratio. This indicates clearly that *BOP* function is conserved between monocots and dicots. Recently, partial defects in ligule development were reported in a single *BOP* mutant of barley, however, the sheath:blade ratio is not affected^34^. In addition, in the maize *bop* mutant, expression of *BOP* genes in the base of the leaf sheath, closely resembling that in rice, has been reported, although leaf development was not obviously affected^35^. This suggests conservation of *BOP* function among grass species. Homology between monocots and dicots in terms of the organs in a leaf has been an issue of debate^56^^,^^57^. One explanation is that the stipule is the homologous organ of the sheath^40^. Indeed, in the mutant of the *COCHLEATA* (*COCH*) gene, an ortholog of *BOP* in pea, the stipule becomes reduced or absent^31^. Alternatively, a comparison of the Arabidopsis *bop* phenotype and rice *bop* mutants indicates that the sheath and the petiole may be equivalent organs.

## Methods

### Gene locus identifier

The locus identification numbers for the *OsBOP* genes are as follows. *OsBOP1* = LOC_Os01g72020, *OsBOP2* = LOC_Os11g04600, *OsBOP3* = LOC_Os12g04410.

### Phylogenetic analysis

BOP/NPR family genes were defined based on both homology searches and the characteristics of the conserved BTB/POZ domains and ankyrin domains in each species, including *Oryza sativa*, *Hordeum vulgare*, *Zea mays*, *Solanum lycopersicum*, *Arabidopsis thaliana*, *Lotus japonicus*, *Medicago truncatula*, *Pisum sativum*, *Amborella trichopoda*, *Selaginella moellendorffii*, *Physcomitrella patens* and *Marchantia polymorpha*. The sequences of all Arabidopsis *BOP* genes were used as queries in BLAST searches. All sequences obtained were aligned using the MUSCLE program. A rooted phylogenetic tree of predicted BOP proteins was constructed from the amino acid sequences of the ankyrin domain using the Maximum Likelihood method, based on the JTT matrix-based model^58^ implemented in MEGA7^59^. The amino acid sequences of the ankyrin domain are shown in Supplementary Fig. 15.

### Plant materials

The Nipponbare cultivar of rice was used throughout the analysis. Rice seeds were germinated at 28 °C in continuous light. Rice plants were grown in growth chambers with daily cycles of 14 h light at 28 °C and 10 h dark at 24 °C. Fluorescent white light tubes were used as the light source. The *osbop1-1*, *osbop1-2*, *osbop1-3*, *osbop2-1*, *osbop2-2*, *osbop2-3*, *osbop3-1*, *osbop3-2* and *osbop3-3* mutants were generated using the CRISPR method with pZH_OsU6gRNA_MMCas9, a vector developed for efficient mutagenesis in rice^42^. The sequences of the guide RNA were designed to target three *OsBOP* genes (Supplementary Fig. 3). All three *OsBOP* genes in the T_0_ generation of the transgenic plants were sequenced to confirm the mutation. Of nine transgenic plants that contained the Target 1 guide RNA, a T_0_ plant was identified carrying heterozygous *osbop1-1*, *osbop2-1*, and *osbop3-1* mutations. T_1_ generation plants obtained from self-crossing of the T_0_ plant were genotyped for the mutation and the introduced gene. The T_1_ plants in which the pZH_OsU6gRNA_MMCas9 construct segregated were used for seed collection. The single, double and triple *osbop* mutants were selected from the descendants and used for analyses. To obtain independent mutations in each *OsBOP* locus, another target guide RNA sequence (Target 2) was used. In the T_1_ generation, plants were selected that displayed a shoot morphology similar to the *osbop1-1 osbop2-1 osbop3-1* triple mutant and the *osbop1-2*/*osbop1-3*, *osbop2-2*/*osbop2-3*, *osbop3-2*/*osbop3-3* mutants were identified. 35s::miR156 and *mSPL14* lines were developed in previous study^52^.

### Vector construction and plant transformation

The construct for target mimicry of miR156 was generated as described in Franco-Zorrilla et al.^60^. The cDNA of *INDUCED BY PHOSPHATE STARVATION1* (*IPS1*) from Arabidopsis was amplified by PCR using the primers, 5ʹ—CACCAAGAAAAATGGCCATCCCCTAGC—3ʹ and 5ʹ—AAGAGGAATTCACTATAAAGAGAATCG—3ʹ. The amplified fragment was introduced into the pENTR/D-TOPO vector (Thermo Fisher Scientific). An artificial mimicry sequence for miR156 was introduced by inverse PCR using the primers, 5ʹ—CTATCTTCTGTCAAGCTTCGGTTCCCCTCGGA—3ʹ and 5ʹ—AAGTGAGCATTTTCTAGAGGGAGATAAAC—3ʹ. The resultant PCR fragment was self-ligated to make the pENTR-MIM156 plasmid. Using the Gateway system (Thermo Fisher Scientific), the insert sequence in pENTR-MIM156 was introduced into the pGWB2 binary vector^61^. The resultant plasmid was transformed into *Agrobacterium tumefaciens* EHA105 by electroporation and used for the transformation of rice. Rice transformation was carried out as described by Nakagawa et al.^62^. Rice calli were co-cultured with the agrobacteria, selected on the selection media containing 50 μg/ml of Hygromycin. From the selected calli, regenerated plants were obtained.

### Real-time PCR

For quantification of the transcripts of *OsBOP*, *OsSPL* and *OsUBQ* genes, the Plant RNA Isolation Mini Kit (Agilent) was used to isolate total RNA. Three biological replicates were prepared for measurement of transcripts. RNA was treated with DNase I and first-strand cDNA was synthesized using SuperScript III reverse transcriptase (Thermo Fisher Scientific). For each sample, PCR reactions were at least duplicated to ensure reproducibility of the results. For quantification of the transcripts, the sequentially diluted cDNA samples were used to generate a standard curve and *Cp* (second derivative method) values were determined by the LightCycler 480 software version 1.5, and exported into an Excel data sheet for analysis. The *Cp* values of the replicated PCR reactions were averaged and used for the analysis. Each mRNA was quantified relative to the level of *OsUBQ* mRNA as an internal control. All the runs were tested with a melt curve analysis to confirm the specificity of PCR amplification. The primer sets used to amplify the transcripts are listed in Supplementary Table 1. For quantification of mature *miR156*, total RNA was extracted using TRIzol reagent (Thermo Fisher Scientific). Real-time PCR analysis of *miR156* was performed using TaqMan MicroRNA Assays (Thermo Fisher Scientific).

### In situ hybridization

In situ hybridizations were performed as described by Kouchi et al.^63^. To generate probes, partial cDNA sequences of *OsBOP1*, *OsBOP2* and *OsLG1*/*OsSPL8* were PCR amplified and cloned into the pENTR/D-TOPO vector (Invitrogen) using primer sets 5ʹ-CACCATGGAGGAAACCCTCAAGTCGCTG-3ʹ and 5ʹ-TCAGGGGAAGCCATGTGGGGAGAA-3ʹ for *OsBOP1*, 5ʹ-CACCATGAGCTCCGAGGACTCGCTCAAATC-3ʹ and 5ʹ-TTATGCGAAGCCATTGGGGAAGTACATGG-3ʹ for *OsBOP2* and 5ʹ-CACCATGGAGGAAACCCTCAAGTCGCTG-3ʹ and 5ʹ-TCAGGGGAAGCCATGTGGGGAGAA-3ʹ for *OsLG1*/*OsSPL8*. To make the antisense probe, in vivo transcription was performed using the linearized plasmid as a template, with the incorporation of digoxigenin (DIG)-UTP by DIG labeling kit (Merck). Tissues were fixed in FAA solution (45% ethanol, 4% formaldehyde, 5% acetic acid) overnight, then gradually dehydrated in a series of ethanol/t-butyl alcohol solutions. After the dehydration, the tissues were infiltrated with Paraplust plus (McCormick Scientific) and then embedded. Microtome sections of 8 µm thickness for embryos and 10 µm thickness for the other tissues were applied onto glass slides treated with Vectabond (Vector Laboratories). The sections on the slides were deparaffinized and rehydrated. The slides were treated with 0.5 µg/ml of proteinase K in proteinase K buffer (100 mM Tris-HCl, 50 mM EDTA, pH 7.5) at 37 °C for 30 min, subsequently fixed in 4% paraformaldehyde in PBS buffer for 10 min. Then the slides were incubated in acetic anhydride solution (0.5 % (v/v) acetic anhydride, 100 mM triethanolamine) for 10 min, followed by incubation in 2× SSPE buffer (20 mM NaH_2_PO_4_, 0.3 M NaCl, 2 mM EDTA, pH 7.4) for 5 min twice. Hybridization was carried out at 55 °C in hybridization buffer (50% Formamide, 0.3 M NaCl, 1× TE, 1× Denhardt’s solution, 10% Dextran sulfate, 1 mg/ml tRNA from yeast, 0.5 mg/ml poly(A)) with the probes overnight. After the hybridization, the slides were washed in 4× SSC (0.6 M NaCl, 6 mM sodium citrate, pH 7.0) at 60 °C and then treated with 50 µg/ml of RNase A (Merck) in RNase buffer (10 mM Tris-HCl, 500 mM NaCl, 1 mM EDTA) at 37 °C for 30 min. After the sections were washed in 0.5× SSC (75 mM NaCl, 0.75 mM sodium citrate, pH 7.0) at 60 °C for 2 h, the sections were incubated in blocking solution (25% Goat serum, 0.5% tween 20, 0.25 M Maleic acid, 0.33 M NaCl) at 30 °C for 30 min, and then incubated with 1/1000 diluted Anti-Digoxigenin-AP in Buffer 1 (0.3 M Maleic acid, 0.44 M NaCl, pH 7.5) at 30 °C for 1 h. The slides were washed with the Buffer 1 and then the signals were developed by incubation with NBT/BCIP (Merck) in Buffer 3 (100 mM Tris-HCl pH9.5, 100 mM NaCl, 50 mM MgCl_2_). After incubation at room temperature for 6–8 h, the signals were observed under an Olympus BX51 microscope.

### Scanning electron microscopy

Small samples frozen in liquid nitrogen or untreated tissues were observed under a scanning electron microscope, JCM-6000 (JEOL) at an accelerating voltage of 15 kV.

### Confocal laser scanning microscopy

The mature embryos in germinating seeds were fixed with 4% PFA overnight. The fixed samples were sliced using a MicroSlicer DTK-1000 (DOSAKA EM). Slices of 150 µm thickness were treated with ClearSee^64^ overnight. The cleared tissues were stained with Direct Red (Sigma-Aldrich), and then imaged under a Zeiss LSM880 confocal microscope.

### Histological analysis

Fresh tissues sliced to 30 µm thickness by a MicroSlicer DTK-1000 (DOSAKA EM) were observed under an Olympus BX51 microscope. For paraffin-embedded sections, samples were fixed with FAA solution overnight at 4 °C. They were dehydrated in a series of ethanol/t-butyl alcohol solutions, then embedded in Paraplast plus (McCormick Scientific). Paraffin-embedded tissue sections of 10 µm thickness were stained with 0.1% Toluidine Blue O.

### Statistics

The Tukey-Kramer test or Dunnett’s test was used for multiple comparisons. For non-parametric variables, the Wilcoxon rank sum test was used. These tests were performed using R 3.3.2. To test statistically significant differences in the results of the real-time PCR experiments, the Student’s *t*-test (two tailed) was performed using Excel unless otherwise indicated. In all statistical tests, *P*-values < 0.05 were considered statistically significant.

## Supplementary information

Supplementary Information PDF

Peer Review

Source Data file

## Data Availability

The locus identification numbers for rice genes described in this article are listed in Supplementary Table 2. A reporting summery for this article is available as a Supplementary Information file. The source data underlying Figs. 3i, 4a–c, 6d–g, 7a,c–f,h, 8b–e and Supplementary Figs 10b–e, 11a-e, 13 are provided as a Source Data file. The raw picture files generated during the current study are available in “figshare” with the identifier “10.6084/m9.figshare.7553186” [doi.org/10.6084/m9.figshare.7553186]. The authors declare that all other data supporting the findings and materials are available from the corresponding authors upon request.
