# Supplementary material for: BLADE-ON-PETIOLE genes temporally and developmentally regulate the sheath to blade ratio of rice leaves
Source: Nat Commun. 2019 Feb 6;10:619. doi: 10.1038/s41467-019-08479-5 (PMC6365560; doi:10.1038/s41467-019-08479-5)
Supplement: Supplementary file 1 — Supplementary Information PDF [file 41467_2019_8479_MOESM1_ESM.pdf]

Supplementary Information for ***BLADE-ON-PETIOLE* genes temporally and developmentally regulate the sheath to blade ratio of rice leaves**

Toriba and Tokunaga et al.

Supplementary Figure 1. Successive leaves in rice.

Supplementary Figure 2. Phylogenetic tree of BOP-related genes.

Supplementary Figure 3. Targeted mutagenesis of *OsBOP* genes using the CRISPR-Cas9 system.

Supplementary Figure 4. Shoot morphology in *osbop* mutants.

Supplementary Figure 5. Defects of *osbop* mutants in ligule (L), auricle (A) and collar (C) development.

Supplementary Figure 6. *in situ* expression analyses of *OsBOP* genes in WT and *osbop* triple mutant.

Supplementary Figure 7. Leaf 2, 3 and 4 in WT and *osbop* mutants.

Supplementary Figure 8. Confocal view of germinating seeds and *OsLG1/OsSPL8* expression.

Supplementary Figure 9. Morphology of the spikelet in *osbop* mutants.

Supplementary Figure 10. Transformed plants containing 35S::*OsBOP1* or 35S::*OsBOP2.*

Supplementary Figure 11. Expressions of *OsSPL* genes and leaf juvenile traits in MIM156 and mSPL14 lines.

Supplementary Figure 12. Expression patterns of the *OsBOP* genes in the mSPL14 line.

Supplementary Figure 13. Leaf juvenile traits in *osbop* single mutants.

Supplementary Figure 14. A model for the regulation of leaf shape by miR156-regulared *OsSPL* genes and *OsBOP* genes.

Supplementary Figure 15. Alignment of the amino acid sequences of the ankyrin domain used for construction of the phylogenetic tree.

Supplementary Table 1. Primers used in real-time PCR

Supplementary Table 2. Rice locus identifiers

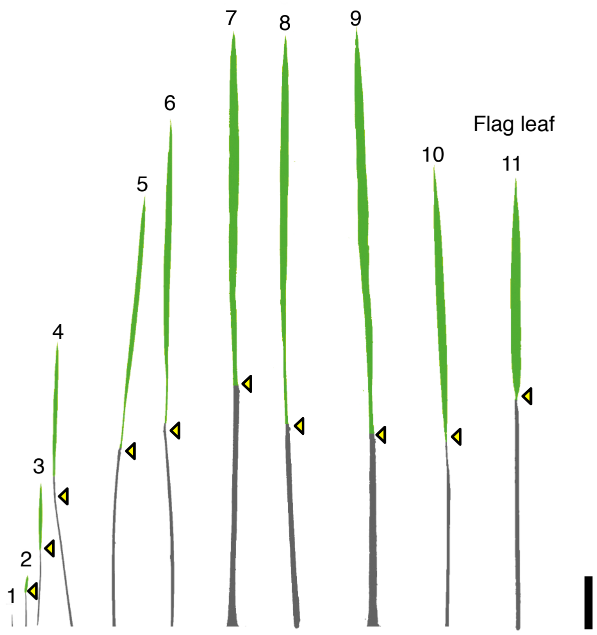

Supplementary Figure 1. | Successive leaves in rice.
The leaf sheath (gray) and the leaf blade (green) develop at the proximal and the distal position, respectively. Yellow arrowheads indicate the boundary between the leaf sheath and blade. The numbers at the top of each leaf indicate the order of their emergence from germination.
Bar: 5 cm.

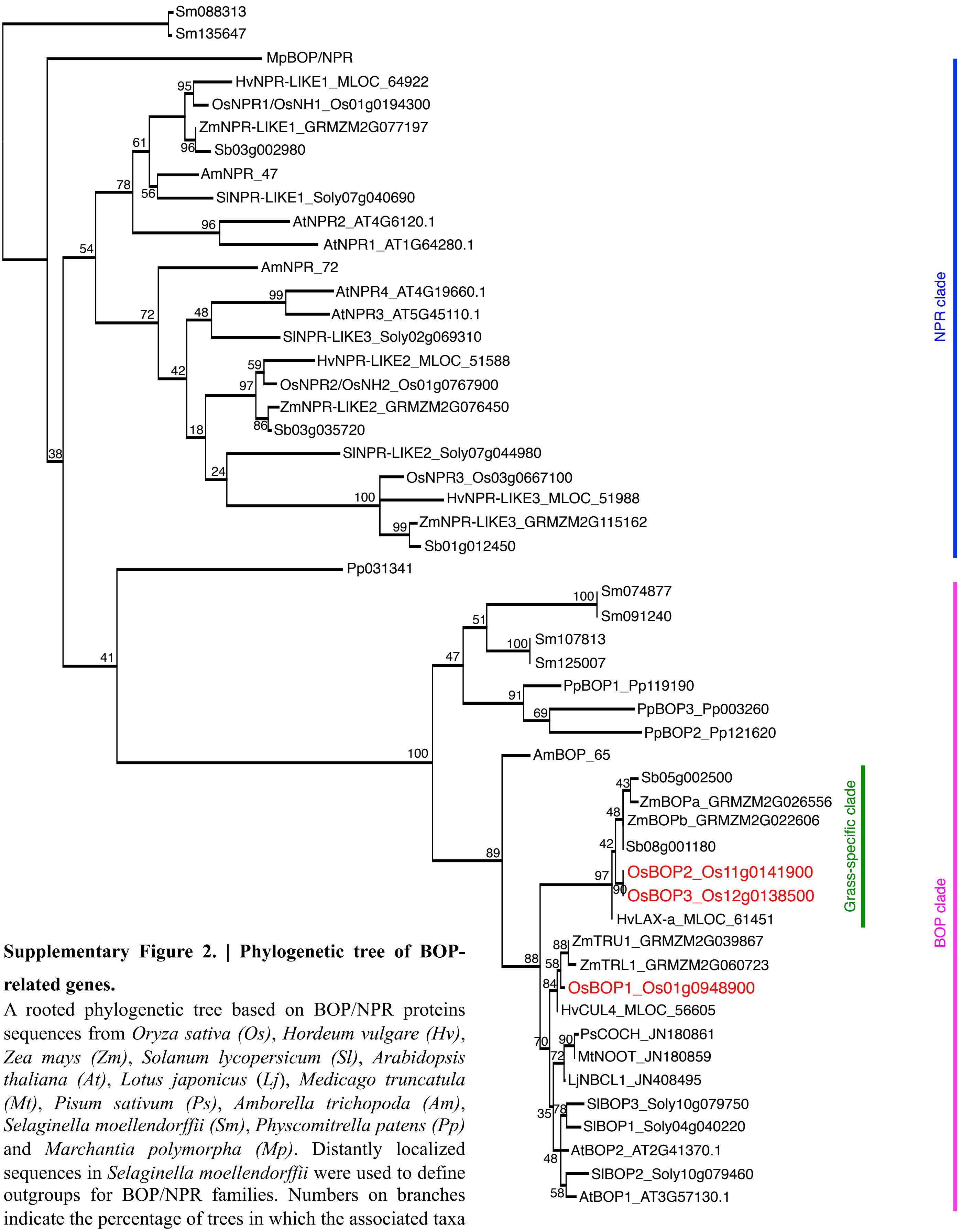

Supplementary Figure 2. | Phylogenetic tree of BOP-related genes.

A rooted phylogenetic tree based on BOP/NPR proteins sequences from *Oryza sativa (Os)*, *Hordeum vulgare (Hv)*, *Zea mays (Zm)*, *Solanum lycopersicum (Sl)*, *Arabidopsis thaliana (At)*, *Lotus japonicus (Lj)*, *Medicago truncatula (Mt)*, *Pisum sativum (Ps)*, *Amborella trichopoda (Am)*, *Selaginella moellendorffii (Sm)*, *Physcomitrella patens (Pp)* and *Marchantia polymorpha (Mp)*. Distantly localized sequences in *Selaginella moellendorffii* were used to define outgroups for BOP/NPR families. Numbers on branches indicate the percentage of trees in which the associated taxa clustered together (percentage of 1000 replicates).

a

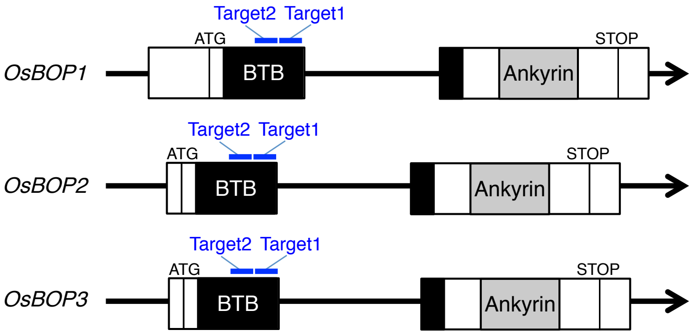

b

```
                              Target1                PAM
                        GTCGAGGGCGAGGTCGACGGCGG
OsBOP1  WT      5' -GTGTCGAGGGCGAGGTCGACGGCGGCGGCGCAGTGGGT-3'
        bop1-1  5' -GTGTCGAGGGCGAGGTCGAACGGCGGCGGCGCAGTGGGT-3'

OsBOP2  WT      5' -GTGTCGAGGGCGAGGTCGACGGCGGCGCCGCACCGCGT-3'
        bop2-1  5' -GTGTCGAGGGCGAGGTCCCTCGGCGGCGCCGCACCGCGT-3'

OsBOP3  WT      5' -GTGTCGAGGGCGAGGTCGACGGCGGCGCCGCACCGCGT-3'
        bop3-1  5' -GTGTCGAGGGCGAGGTCGAACGGCGGCGCCGCACCGCGT-3'
```

c

```
                             Target2                 PAM
                        GTCCTGCAGTTCCTGTACAGCGG
OsBOP1  WT      5' -TGCTCCTCCAGTTCCTGTACAGCGGTCAGGTGTCGCTG-3'
        bop1-2  5' -TGCTCCTCCAGTTCCTGTAACAGCGGTCAGGTGTCGCTG-3'
        bop1-3  5' -TGCTCCTCCAGTTCCTGTATCAGCGGTCAGGTGTCGCTG-3'

OsBOP2  WT      5' -TCGTGCTGCAGTTCCTGTACAGCGGGCAGGCGTCGGTG-3'
        bop2-2  5' -TCGTGCTGCAGTTCCTGTACCAGCGGGCAGGCGTCGGTG-3'
        bop2-3  5' -TCGTGCTGCAGT-------------------------TG-3'

OsBOP3  WT      5' -TCGTGCTGCAGTTCCTGTACAGCGGGCAGGCGTCGGTG-3'
        bop3-2  5' -TCGTGCTGCAGTTCCTGTACCAGCGGGCAGGCGTCGGTG-3'
        bop3-3  5' -TCGTGCTGCAGTTCCTGTAACAGCGGGCAGGCGTCGGTG-3'
```

Supplementary Figure 3. | Targeted mutagenesis of *OsBOP* genes using the CRISPR-Cas9 system.
(**a**) The target position is shown above the structure of the *OsBOP* genes. Target sites 1 and 2 are located in sequences encoding BTB/POZ domain. Black and gray blocks indicate sequence regions corresponding to BTB/POZ domain and Ankyrin repeat, respectively.
(**b**, **c**) *OsBOP1*, *OsBOP2* and *OsBOP3* mutant alleles used in this study. The sequences of the wild-type and the mutant alleles are aligned with the target sites. The blue font shows the gRNA target sequence. The red font denotes mutations such as insertion, replacement or deletion of bases.

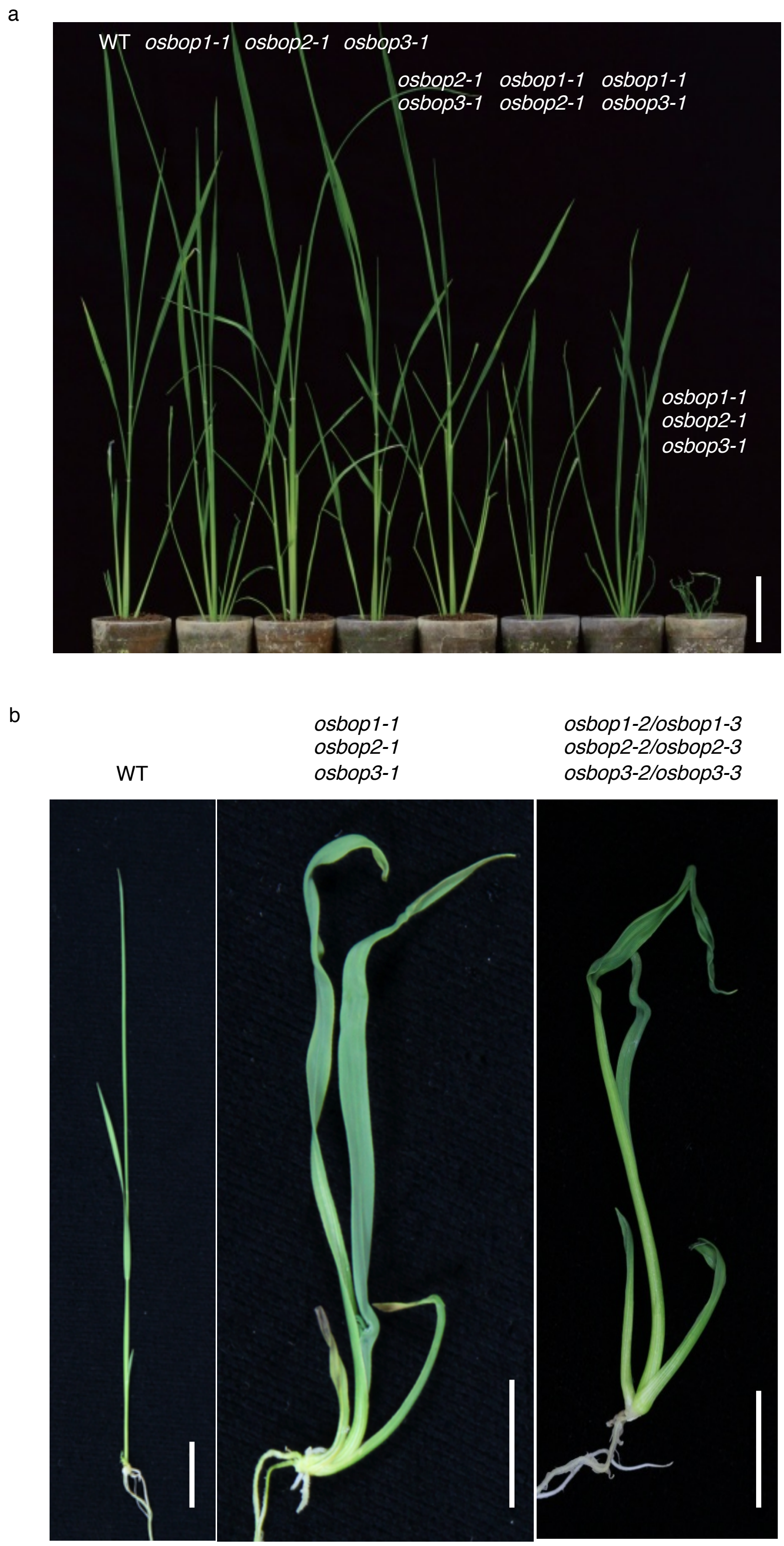

Supplementary Figure 4. | Shoot morphology in *osbop* mutants.

(**a**) Shoots in the WT, *osbop* single, double and triple mutants.

(**b**) Seedlings in the WT, *osbop1-1 osbop2-1 osbop3-1* and the bi-allelic triple mutant.

Bars: 5 cm (**a**), 1 cm (**b**).

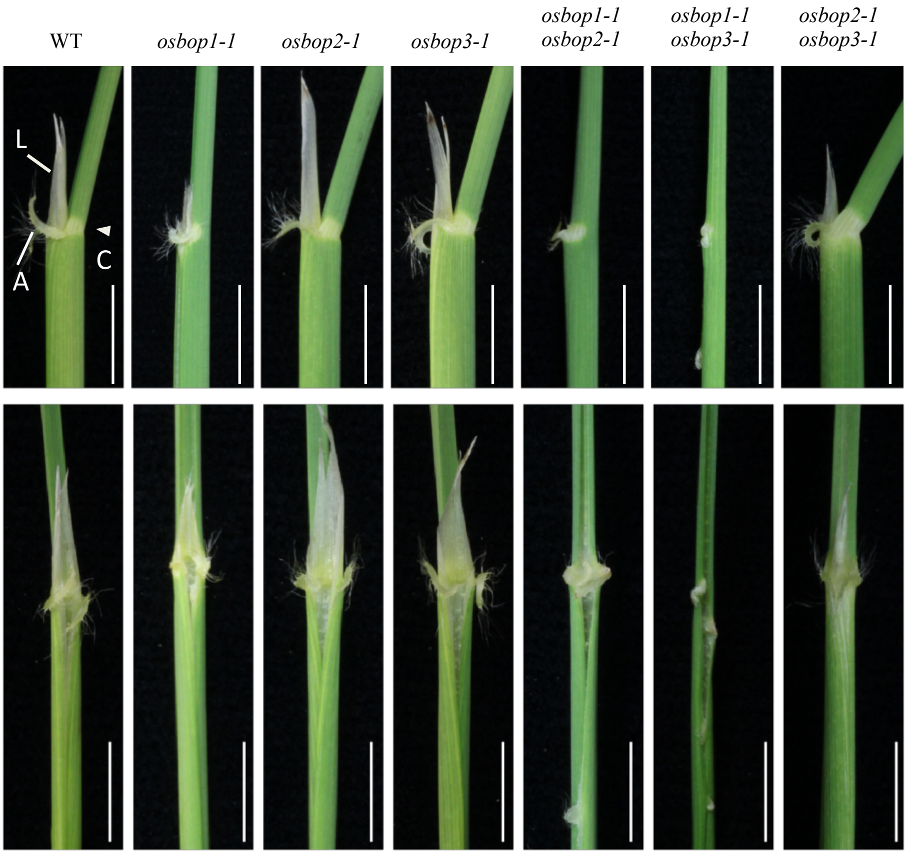

Supplementary Figure 5. | Defects of *osbop* mutants in ligule (L), auricle (A) and collar (C) development.
The boundary regions between the leaf blade and sheath in each genotype are shown.
Bars: 5 mm.

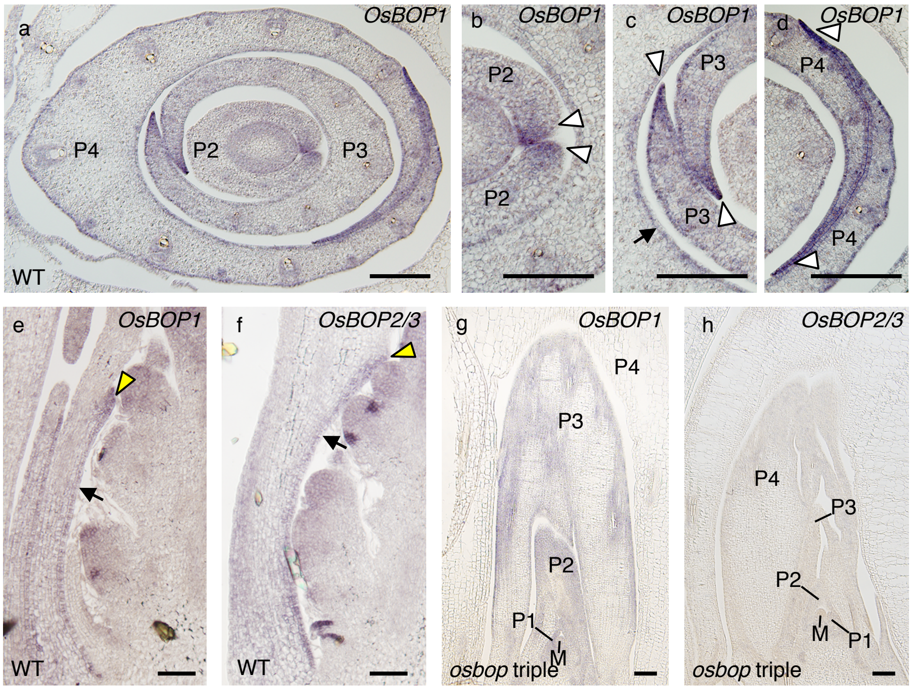

Supplementary Figure 6. | *in situ* expression analyses of *OsBOP* genes in WT and *osbop* triple mutant.

(**a**) Localization of *OsBOP1* mRNA in a cross-section of the lower part of the WT shoot. (**b-d**) Close-up views of the marginal regions in P2 (**b**), P3 (**c**) and P4 (**d**) leaves.White arrowheads indicate leaf margins. (**e**,**f**) Localization of *OsBOP1*(**e**) and *OsBOP2/3* (**f**) mRNA in a developing flag leaf. Black arrows indicate an epidermis of leaf sheath. Yellow arrowheads indicate ligule primordia. (**g**,**h**) A longitudinal section of *osbop* triple shoots hybridized with the *OsBOP1* antisense probe (**g**) or *OsBOP2/3* antisense probe (**h**). M, shoot apical meristem. Bars: 100 μm.

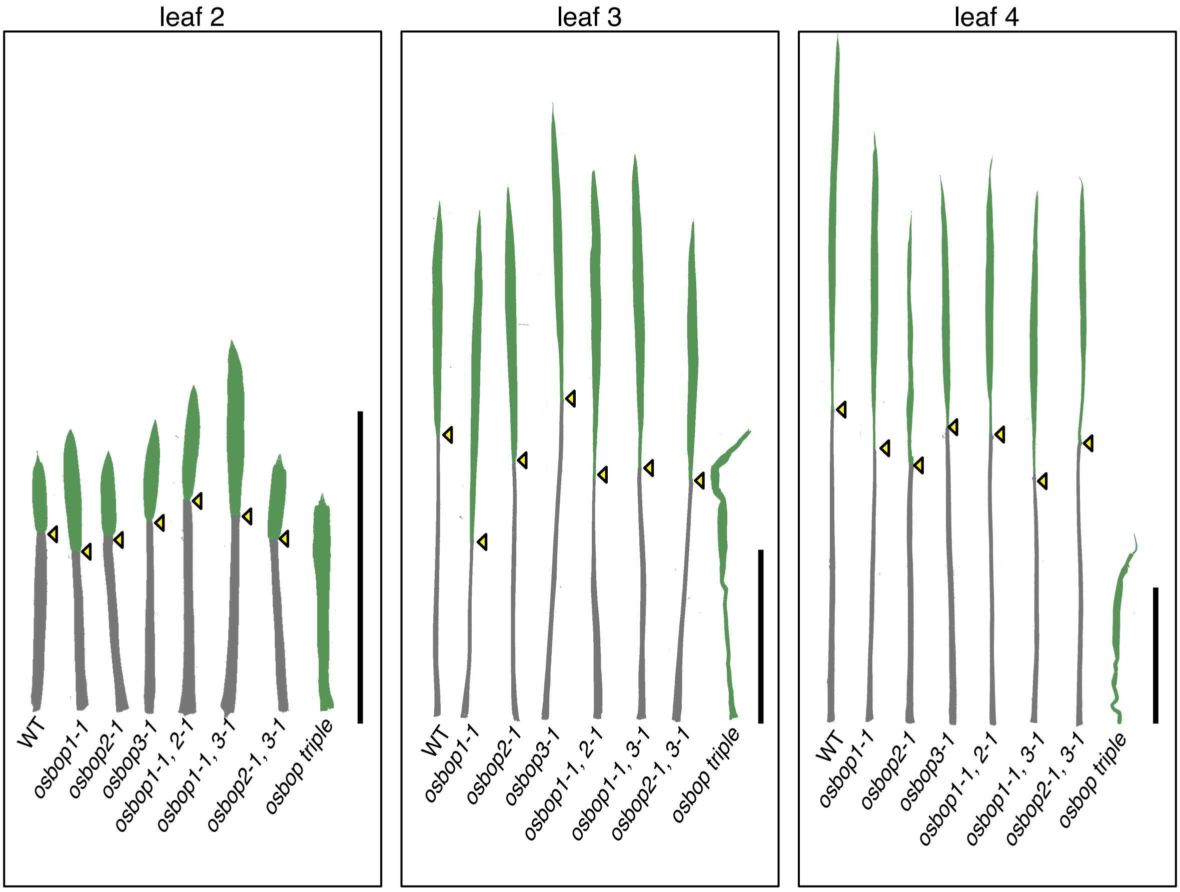

Supplementary Figure 7. | Leaf 2, 3 and 4 in WT and *osbop* mutants.
The shapes of leaf sheath (gray) and leaf blade (green) are shown. Yellow arrowheads indicate the boundary between the leaf sheath and blade. Bars: 5 cm.

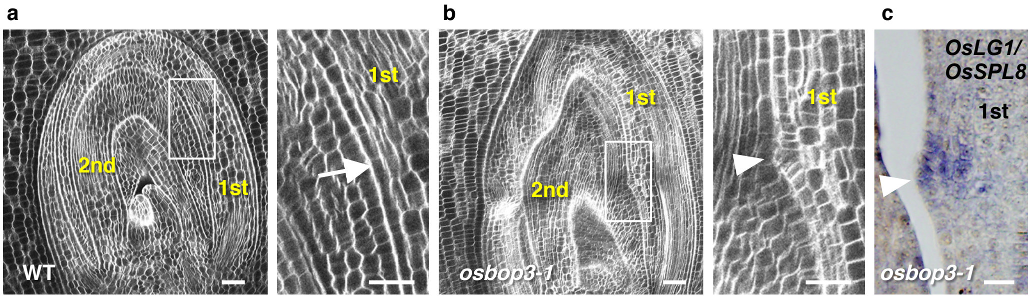

Supplementary Figure 8. | Confocal view of germinating seeds and *OsLG1/OsSPL8* expression.
(**a**) A WT shoot in the seed after 12 h imbibition. A close-up view of the white square in (**a**) is shown in the panel immediately to the right. A white arrow indicates the epidermal cell layer in the first leaf.
(**b**) An *osbop3-1* shoot in the seed after 12 h imbibition. A close-up view of the white square in (**b**) is shown in the panel immediately to the right. A white arrowhead indicates the initiating ligule from the epidermal cell layer in the first leaf.
(**c**) Localization of *OsLG1/OsSPL8* mRNA in the first leaf in the *osbop3-1* seed after 24 h imbibition. A white arrowhead indicates the initiating ligule from the epidermal cell layer in the first leaf.
Bars: 20 μm

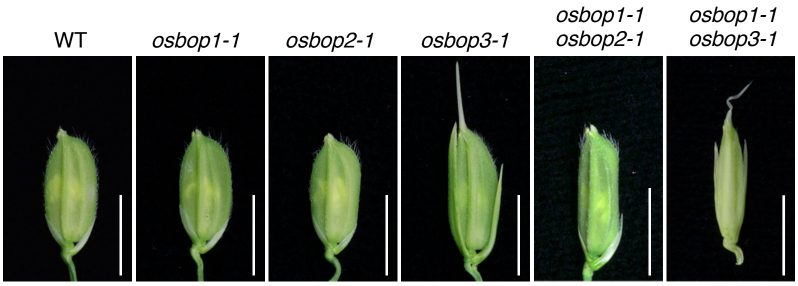

Supplementary Figure 9. | Morphology of the spikelet in *osbop* mutants. Bars: 5 mm.

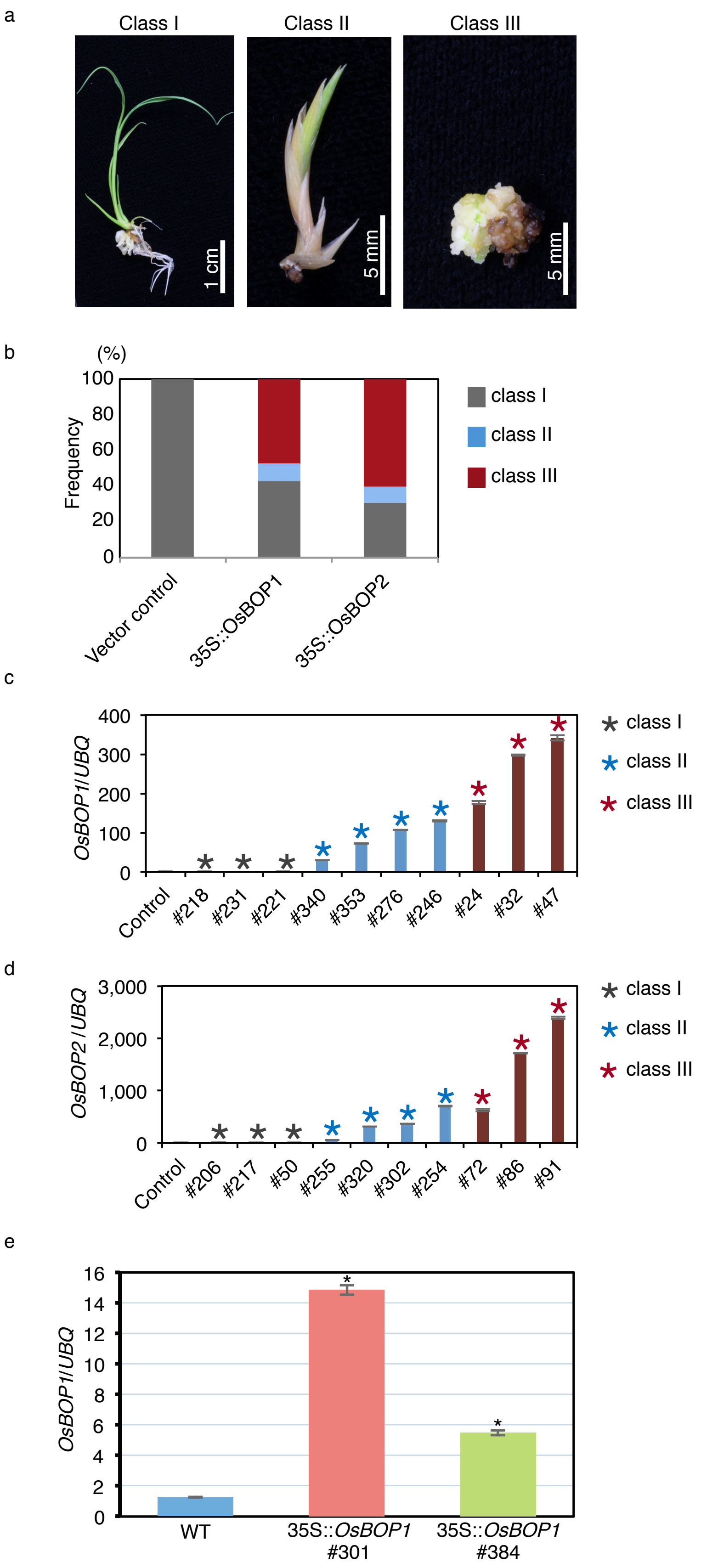

Supplementary Figure 10. | Transformed plants containing 35S::*OsBOP1* or 35S::*OsBOP2*.
(**a**) Classification of the transgenic plants. Class I: normal looking plantlet, Class II: dwarf plantlet containing only a leaf sheath, Class III: no plants regenerated. (**b**) Frequency of production of three types of transformants. n = 37 for Vector control, n = 144 for 35S::*OsBOP1* and n = 158 for 35S::*OsBOP2*. (**c**) Correlation between the expression level of *OsBOP1* and the phenotypic classification. Error bars indicate standard error of three technical replicates. (**d**) Correlation between the expression level of *OsBOP2* and the phenotypic classification. Error bars indicate standard error of three technical replicates. (**e**) Relative expression levels of *OsBOP1* in T1 plants of 35S::*OsBOP1* #301 and #384. The expression level of the WT is set as 1.0. Error bars indicate standard error (n = 3). * indicates a significant difference compared to the WT (Student's t test, $P < 0.05$). Source data are provided as a Source Data file.

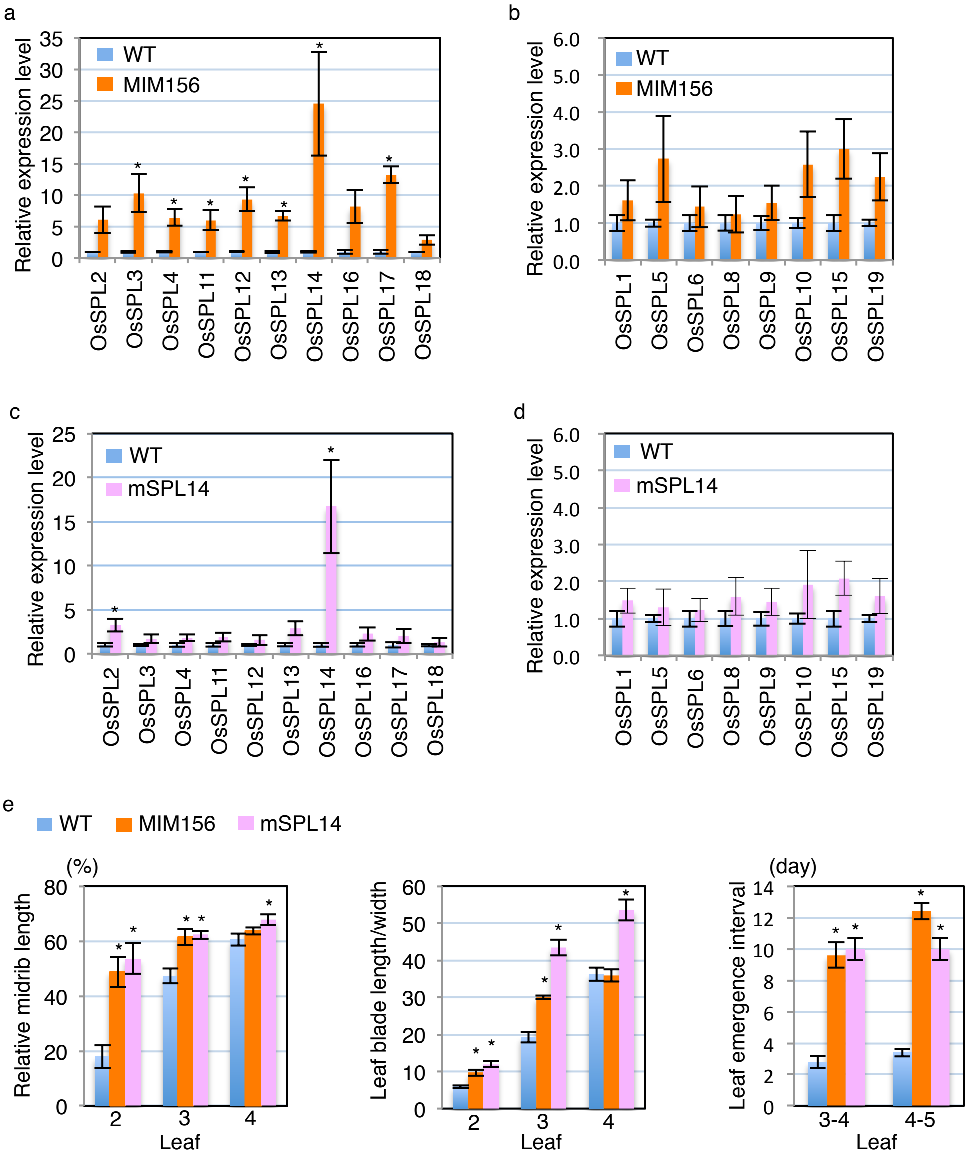

Supplementary Figure 11. | Expressions of *OsSPL* genes and leaf juvenile traits in MIM156 and *mSPL14* lines.
(**a**,**b**) Relative expression levels of miR156-targeted *OsSPL* genes (**a**) and non-targeted *OsSPL* genes (**b**) in the first leaf in the MIM156 line. Each leaf was collected when it was emerging from the coleoptile. The expression level of each gene was normalized to *OsUBQ* expression, and relative expression levels in the WT were set as 1.0. Error bars indicate standard error (n = 3 biologically independent samples). Among all *OsSPL* genes examined, *OsSPL3*, *OsSPL4*, *OsSPL11*, *OsSPL12*, *OsSPL13*, *OsSPL14* and *OsSPL17* show statistically higher expression levels in the MIM156 line indicated by "*" on the graph (Student's t test, $P < 0.05$).
(**c**.**d**) Relative expression levels of miR156-targeted *OsSPL* genes (**c**) and non-targeted *OsSPL* genes (**d**) in the first leaf in the *mSPL14* line. Each leaf was collected when it was emerging from the coleoptile. The expression level of each gene was normalized to *OsUBQ* expression, and relative expression levels in the WT were set as 1.0. Error bars indicate standard error (n = 3 biologically independent samples). Among all *OsSPL* genes examined, *OsSPL2* and *OsSPL14* show statistically higher expression levels in the *mSPL14* line indicated by "*" on the graph (Student's t test, $P < 0.05$). (**e**) The ratio of midrib length/blade length (left), the ratio of leaf blade length/width (middle) and the length of leaf emergence interval (right). Error bars indicate standard error (n = 5). "*" indicates a statistically significant difference compared to the WT (Dunnett's test, $P < 0.05$ for relative midrib length and leaf blade length/width; Wilcoxon rank sum test, $P < 0.05$ for leaf emergence interval). Source data are provided as a Source Data file.

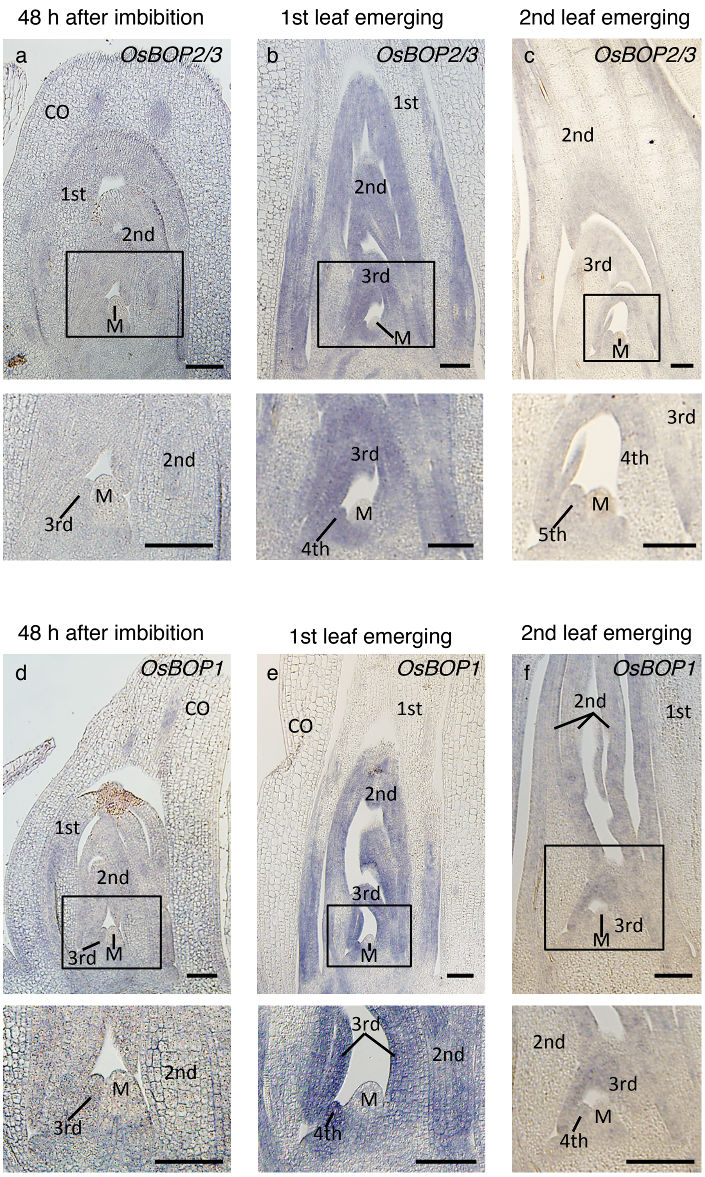

Supplementary Figure 12. | Expression patterns of the *OsBOP* genes in the mSPL14 line. Localization of *OsBOP2/3* mRNA (**a-c**) and *OsBOP1* mRNA (**d-f**) in germinating shoots 48 h after imbibition (**a**,**d)**, at the emergence of the first leaf (**b**,**e**) and at the emergence of the second leaf (**c**,**f**). A close-up view of the white squares in (**a-f**) is shown immediately below the panel. CO, coleoptile; M, shoot apical meristem. Bars: 100 μm.

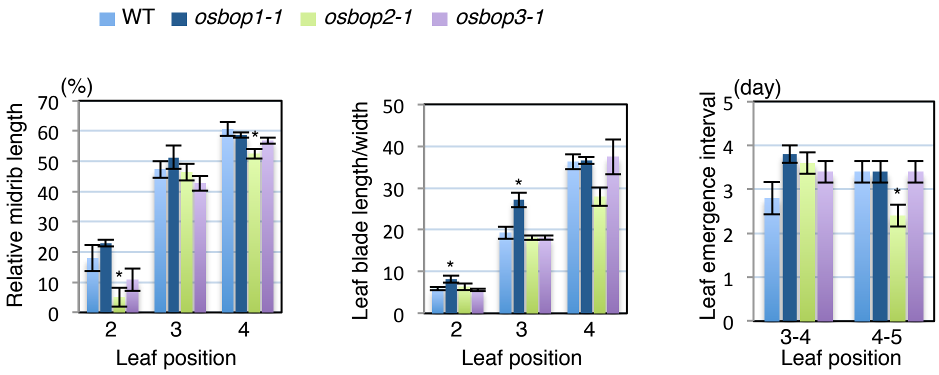

Supplementary Figure 13. | Leaf juvenile traits in *osbop* single mutants.
The ratio of midrib length/blade length (left), the ratio of leaf blade length/width (middle) and the length of leaf emergence interval (right). Error bars indicate standard error (n = 5 biologically independent samples). "*" indicates statistically significant difference compared to WT (Dunnett's test, $P < 0.05$ for relative midrib length and leaf blade length/width; Wilcoxon rank sum test, $P < 0.05$ for leaf emergence interval). Source data are provided as a Source Data file.

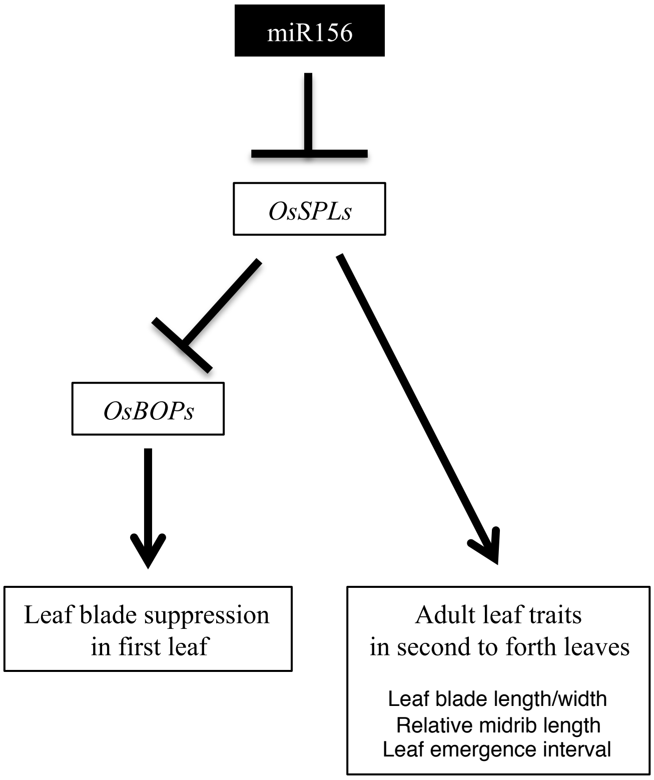

Supplementary Figure 14. | A model for the regulation of leaf shape by miR156-regulared *OsSPL* genes and *OsBOP* genes.

We propose distinct pathways controlled by miR156-regulated *OsSPL* genes in regulation of leaf shape in juvenile vegetative phase. One pathway explains the suppression of leaf blade via the regulation of the expressions of *OsBOP* genes in the first leaf. The other pathway represents the expression of adult leaf traits in the second to fourth leaves.

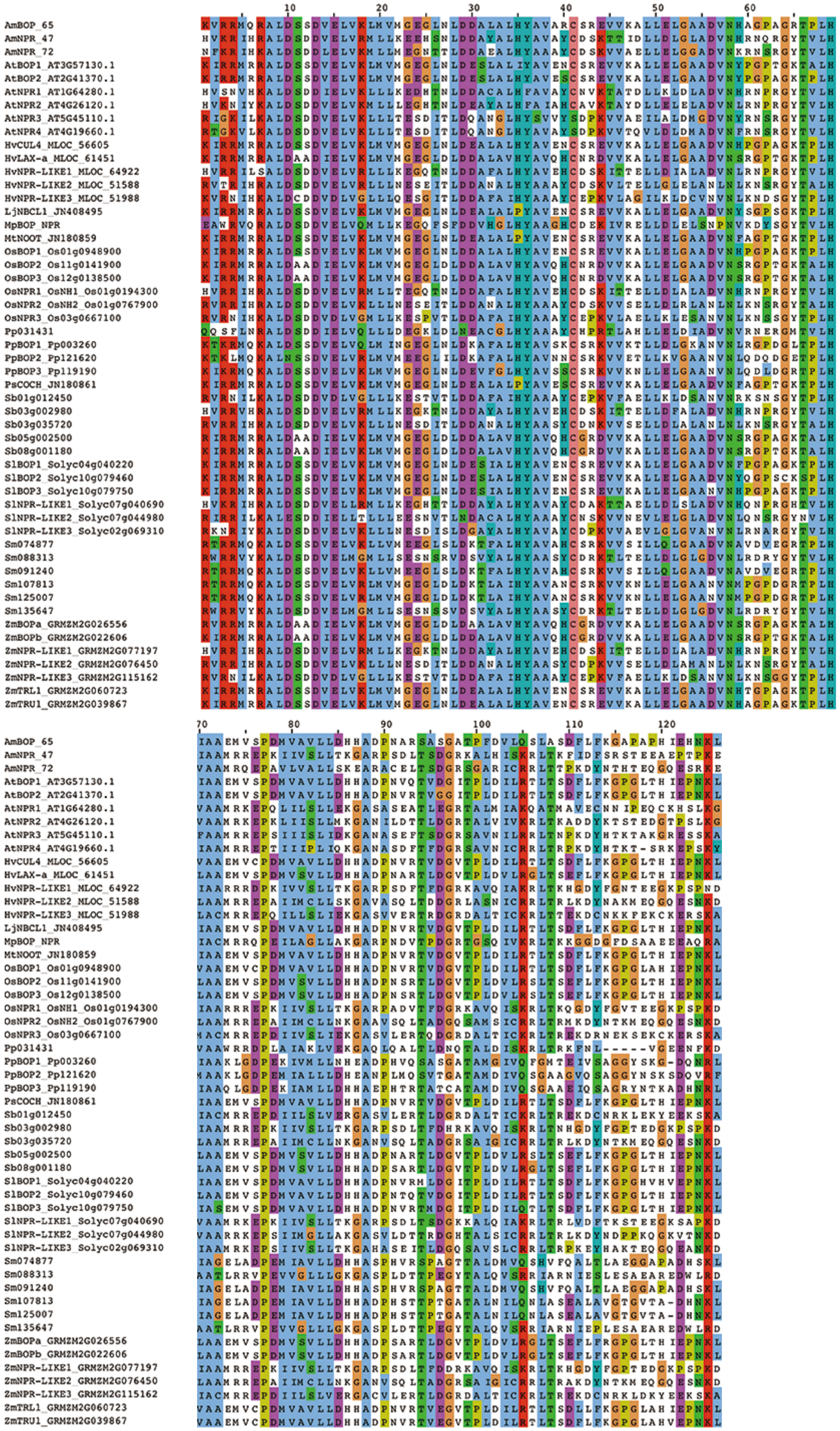

Supplementary Figure 15. | Alignment of the amino acid sequences of the ankyrin domain used for construction of the phylogenetic tree.

Supplementary Table 1. Primers used in real-time PCR

gene name	forward primer (5'-3')	reverse primer (5'-3')
OsBOP1	TGAGCTTGGACAACAGGATG	CATGTGGGGAGAACAACGAC
OsBOP2/3	AGCCTGACGATGAACTCGAC	GAAGCCATTGGGGAAGTACA
OsSPL1	CCTCAGCAGCTGGGAATCAA	TTCCGAGCGTAATCCTCCGG
OsSPL2	CCACTTCCCTTTCCTGACGAC	GAGGCTGTGGGATGGTGATC
OsSPL3	CAAGGCATGGAAGCTTCTGC	GCTGATCAAGGGACAAGCCT
OsSPL4	GCCTGTGAATTGGATGACGC	CCGGACGCATCTTTAACTGC
OsSPL5	CCAGCATCGCTAGCTCCTAC	GGTGGTCTGCAAGCTCCTG
OsSPL6	GATGGTGTGAAGCCTGGTGA	CGTCGAGTTGGGGTACATC
OsSPL8	GAACGCAGGCATTGGGGAC	GGAATAAGCATGAGCCAGACG
OsSPL9	CACACAGGCAATGCTGTCAC	ACCACCTGGAAAAGCACCAA
OsSPL10	CCATCACCAGCAAGACCACA	CTGATACCTCGCCGTTGGTC
OsSPL11	GCACTAGATGTTCAGCGTGC	CCTGAGGCTCTACTGCTTGA
OsSPL12	CAGGCAATGTTGTGTCGCAT	CCCAGGAATCGCTTGACAGA
OsSPL13	GGAGTTCGACGACGCGAA	GATCTGGAACGGCGGGTT
OsSPL14	CCAAATCTCCCTCCAGGTGG	GCGGCACTGTGGGTAGTAGTA
OsSPL15	GGGAGCAGAGAAACCCTGAC	ATAGCGTCCCCATCTCACCT
OsSPL16	CCACAGCCAGATCCCATGAA	GCGAACGGTGAGAATCTTGC
OsSPL17	CGACACCTGGACCTCTTTCT	TCCCTCACACTGCTTGGAAC
OsSPL18	GAAAACGCTTGGATGGGCAC	AAGCTGCCAGAACTCATGCT
OsSPL19	CCAACATTACCACAACCCGC	ATGCAGCGACGATGACAGAA
UBQ	AACCAGCTGAGGCCCAAGA	ACGATTGATTTAACCAGTCCATGA

Supplementary Table 2. rice locus identifiers

gene name	locus ID
OsBOP1	LOC_Os01g72020
OsBOP2	LOC_Os11g04600
OsBOP3	LOC_Os12g04410
OsUBQ	LOC_Os03g13170
OsSPL1	LOC_Os01g18850
OsSPL2	LOC_Os01g69830
OsSPL3	LOC_Os02g04680
OsSPL4	LOC_Os02g07780
OsSPL5	LOC_Os02g08070
OsSPL6	LOC_Os03g61760
OsSPL7	LOC_Os04g46580
OsSPL8	LOC_Os04g56170
OsSPL9	LOC_Os05g33810
OsSPL10	LOC_Os06g44860
OsSPL11	LOC_Os06g45310
OsSPL12	LOC_Os06g49010
OsSPL13	LOC_Os07g32170
OsSPL14	LOC_Os08g39890
OsSPL15	LOC_Os08g40260
OsSPL16	LOC_Os08g41940
OsSPL17	LOC_Os09g31438
OsSPL18	LOC_Os09g32944
OsSPL19	LOC_Os11g30370
