# Supplementary material for: BLADE-ON-PETIOLE genes temporally and developmentally regulate the sheath to blade ratio of rice leaves
Source: Nat Commun. 2019 Feb 6;10:619. doi: 10.1038/s41467-019-08479-5 (PMC6365560; doi:10.1038/s41467-019-08479-5)
Supplement: Supplementary file 2 — Peer Review [file 41467_2019_8479_MOESM2_ESM.pdf]

Journal peer review information: *Nature Communications* thanks Jia-Wei Wang, and the other anonymous reviewer(s) for their contribution to the peer review of this work. Peer reviewer reports are available.

Reviewers' comments:

Reviewer #1 (Remarks to the Author):

The paper by Kyozuka brings considerable insight into leaf development. By examining triple bop mutants in rice they identify a clear determinant of proximal identity. Grass leaves have a proximal sheath, a distal blade and a junction region containing the ligule and auricle. The triple bop mutant is not only liguleless, but loses most of its sheath identity. A single bop mutant has been described in barley that is slightly liguleless but still has clear blade/sheath boundaries. This triple mutant is really striking. Intriguingly, the sheath-less bop leaves also fail to wrap the stem as in normal sheath. Thus, the loss of sheath identity is more than just changes in epidermal patterning.

Another fascinating phenotype is the reappearance of blade in the first leaf. Wild-type rice first leaves are just sheath. These phenotypes strongly support the idea that BOP genes repress blade and promote sheath.

Their in situs show expression in the margins of leaves starting at P2. They also show expression in the epidermis of P4 leaves. I think an appropriate control would be a mutant to show there is no transcript. They say the expression goes up to the ligule, but it is hard to see this.

Because of the strong effect on the first leaf, which is made in the seed, they look early in development at embryos. They see strong BOP expression in embryos. They also look at shoots after 2 days of imbibition and time points later on. BOP2/3 and BOP1 are strongly expressed in shoots throughout the youngest leaves (but not the meristem). Expression is dampened at 1st leaf emerging (1 week later or so??) and very dampened at 2nd leaf emerging. (How does this compare with the timing for figure 2, which is 6th leaf stage?). Interesting that it doesn't seem to be a proximal marker as it is expressed through out the P3 or P4 primordia. Is this pattern different because of the microRNA control? At the second leaf emerging stage the BOP expression is very low and just seen at the base of the 3rd leaf. Is this a sheath domain? I think a little more clarity on the expression domains through time and location would help.

They also look at the spikelets. BOP2/3 is expressed in the leaf like organs of the spikelet (nice in situs) and the leaf-like organs are elongated.

They then overexpress BOP1 and BOP2. Only weak expressors survived and they were shoots with short blades. Nice result.

Is the ligule expression and function truly independent as they state?

Because of the change in expression levels of the BOPs from embryos to later stages, they follow the effect of miR156, which is expressed at high levels in the first leaf. They make miM156 and mSPL14 plants and see a blade forming in the first leaf, similar to the bop mutant. These results place the SPL14 upstream of bop. They crossed the mSPL14 plant to the 35S:BOP and saw that the BOP was epistatic, again placing BOP downstream.

Minor comments FIg 2 panel B. is it OsBOP2/ or should it be 2/3?
Page 7. Line 129. Do they mean sheath when they say leaf?
Page 8. Line 135. I think they mean proximal here. Don't the BOPs suppress blade in the proximal region (where there is sheath)?
I don't know if the "knots"adds anything in Fig 6h-i. It is hard to make out what we are looking at.
Figure 7a. They state leaf position, but isn't it leaf 1, leaf 2, etc? I think of position as something that could occur throughout development.
The upper right corner panels of Figure 6 were not mentioned. They seem quite nice – so please

discuss them.

Reviewer #2 (Remarks to the Author):

1. The title is not attractive. It is already known that BOP genes are involved in leaf proximal-distal patterning in Arabidopsis (for example, Ha et al., Genetics, 2010). What' new here is that the temporal change of BOP levels, probably by miR156-SPL aging module, contributes to the heteroblastic leaf trait (the gradual change in sheath/blade ratio) in rice. Therefore, a new title will be better.
2. The phenotypic analyses were of high quality. For the expression analyses, the qRT-PCR results (Fig. 7e and j) are not informative enough. I would like to see a time-course analyses of BOP transcripts by in situ, from leaf 1 to leaf 4, in both wild-type and miR156ox/spl14 mutant background. These results will help us to know how miR156 precisely regulates the BOP expression. Does miR156 spatially regulates BOP？or just quantitatively?
3. I think the Fig 5 is not essential, not closely related to the topic of the manuscript. Authors could move it to the supplemental materials.
4. The introduction: the summary of the miR156-SPL pathway and juvenile-to-adult phase transition in rice could be expanded since the temporal change of sheath/blade ratio is the highlight of the manuscript. For example, the rice COP1/PPS has been shown to play a role in juvenile-to-adult phase transition in rice (Tanaka, TPC, 2011). Line 60-61: there are no references about the role of miR156 in juvenile-to-adult phase transition. There are some suitable review papers from Scott Poethig, Jia-Wei Wang, or Markus Schmid lab.

In summary, the manuscript is interesting and well written. Most of the experiments were performed at a high standard. The emphasis of the temporal regulation of sheath/blade ratio by miR156-BOP will further strengthen the novelty of the manuscript.

Reviewer #3 (Remarks to the Author):

This manuscript describes a role for rice BLADE-ON-PETIOLE genes in proximal-distal leaf patterning. Grass leaves consist of a proximal sheath and a distal blade separated by boundary organs consisting of an auricle and ligule. CRISPR-Cas9 directed mutations were induced to generate single, double, and triple mutations in OsBOP1, OsBOP2, and OsBOP3. 35S:OsBOP1 transgenic plants were also generated. Analysis of these loss-and gain-of-function mutants showed that BOPs are collectively required for the formation of boundary structures as well as promote sheath cell identity and repress blade development, particularly in the first leaf where BOPs expression levels are highest. The authors provide convincing evidence that this expression pattern is under the influence of miR156 and SPL family genes that control age-dependent changes associated with phase transition in plants. These characterizations of BOP activity align well with roles in eudicot plants and regulation by miR156/SPLs for leaf shaping is new and interesting. This well-written paper sheds light on how monocot leaves are patterned and illustrates the strong potential of CRISPR/Cas9 technology for reverse genetic studies in crop plants. The weakness is that the mechanism of SPL regulation of BOPs remains unclear and no link is made to domestication traits in rice.

Minor comments:
1. Abstract, temporal changes in expression of OsBOPs CORRELATE with developmental changes in the sheath:blade ratio. We cannot say they are responsible without additional data. Further, it may be premature to conclude the OsBOPs are the main regulators of proximal-distal patterning rice leaf development since loss-of-function mutations in OsBOPs mainly affect the first leaf.

2. A developmental series of leaves for osbop combination mutants showing leaf shape changes

should be included (to compare to Supplemental Figure 1 which shows pattern for wild-type leaves). Are blades in the double and triple osbop mutants wider than in WT? There do not appear to be any significant changes in the sheath length according to Figure 3. It is difficult to process all these dimensions without a leaf series.

3. Supplementary Figure 2. The phylogenetic tree is constructed using a neighbor joining method, which is considered less reliable compared to bayesian/maximum likelihood trees for inferring evolutionary histories. The tree should be redrawn. Related to that, the authors do not explain the significance of a grass-specific clade for BOPs. Is this clade predicted to have a specific function in monocots that sets them apart from eudicot BOPs?

4. The authors propose that OsSPLs repress OsBOP expression allowing blades to develop in leaf 2 and higher (model/text). How then do we account for rice spl8/liguleless1 mutants that lack boundary structures similar to osbop loss-of-function mutants?

5. The in situ pictures do not make it clear if OsBOP expression is enriched in the sheath relative to the blade on individual leaves. Any evidence that KNOX genes are ectopically expressed in the sheath region of rice leaves as shown previously for Atbop1 bop2 mutants?

6. Supplemental Figure 1. Age of plants is not indicated. Yellow arrowheads and number labels are missing on this figure.

7. Figure 3. Arrow color indicating the ectopic leaf that forms on the sheath should be clearly identified in the Figure Legend. Figure 3b. The bar graph is missing the triple mutant. Figure 3n. Square of close-up region should be black (not yellow).

8. Line 188: These data imply….

9. Figure 4bc, Figure 7, Supplementary Figure 10. Show significant differences on the graphs with asterisks.

10. Figure 2. The labels for OsBOP1 in (a) and OsBOP2/3 in (b) have slipped off the panels. Also, panel labels for e,f,g are outside the panels and whereas the rest of the letters are inside.

11. Supplementary Figure 8. Scale bar for osbop2-1 is slightly thinner than the rest.

12. Supplementary Figure 3. Are the guide RNA target sites located in the BTB/POZ domain? Indicate.

13. Figure 5d. Why is the OsBOP2/3 signal present as punctate dots?

14. BOP knockouts are expected to exhibit defects in flower patterning and abscission. The authors do not specify.

15. Materials and Methods. Plant materials. Growth conditions for the rice plants are not given. Include a greater description of how the double and triple mutants were generated after CRISPR/Cas9 singles were found. How many plants were screened to find the initial mutations? Real-time PCR. Include a greater description of conditions used for qPCR, primer design and validation methods, specific statements about the number of biological replicates and normalization methods. Supplementary Table 1. Add unique identifier numbers for rice genes if possible.

Responses to Reviewers

Reviewer #1 (Remarks to the Author):

The paper by Kyozuka brings considerable insight into leaf development. By examining triple bop mutants in rice they identify a clear determinant of proximal identity. Grass leaves have a proximal sheath, a distal blade and a junction region containing the ligule and auricle. The triple bop mutant is not only liguleless, but loses most of its sheath identity. A single bop mutant has been described in barley that is slightly liguleless but still has clear blade/sheath boundaries. This triple mutant is really striking. Intriguingly, the sheath-less bop leaves also fail to wrap the stem as in normal sheath. Thus, the loss of sheath identity is more than just changes in epidermal patterning.

Another fascinating phenotype is the reappearance of blade in the first leaf. Wild-type rice first leaves are just sheath. These phenotypes strongly support the idea that BOP genes repress blade and promote sheath.

Their in situs show expression in the margins of leaves starting at P2. They also show expression in the epidermis of P4 leaves. I think an appropriate control would be a mutant to show there is no transcript. They say the expression goes up to the ligule, but it is hard to see this.

[Answer] We added *in situ* hybridization data showing that the expression levels of *OsBOP1* and *OsBOP2/3* in *osbop1,2,3* triple mutants are undetectable (see Supplementary Figure S6 g, h, line 154-156, page 9). We also added *in situ* hybridization data showing that the *OsBOP* transcript is localized in the epidermis of leaves between the leaf base and the ligule which shows that the *OsBOP* expression stops at the ligule position (Supplementary Figure S6 e, f). In addition, we modified the text describing the expression pattern to clearly indicate that the expression starts from the base of leaf primordia and expands upward toward the boundary region (line 158-160, 167-170, page 9-10).

Because of the strong effect on the first leaf, which is made in the seed, they look early in development at embryos. They see strong BOP expression in embryos. They also look at shoots after 2 days of imbibition and time points later on. BOP2/3 and BOP1 are strongly expressed in shoots throughout the youngest leaves (but not the meristem). Expression is dampened at 1st leaf emerging (1 week later or so??) and very dampened at 2nd leaf emerging. (How does this compare with the timing for figure 2, which is 6th leaf stage?). Interesting that it doesn't seem to be a proximal marker as it is expressed throughout the P3 or P4 primordia. Is this pattern different because of the microRNA control? At the second leaf emerging stage the BOP expression is very low and just seen at the base of the 3rd leaf. Is this a sheath domain? I think a little more clarity on the expression domains through time and location would help.

[Answer] We modified the text describing the expression patterns of the *OsBOP*s during the early stages of plant development. In order to observe the expression patterns of the *OsBOP*s in each leaf at

a fixed time (e.g. P2, P3), we carefully observed the timing of leaf primordia differentiation from embryogenesis until the fourth leaf differentiation stage, before conducting *in situ* hybridization experiments.

They also look at the spikelets. BOP2/3 is expressed in the leaf like organs of the spikelet (nice in situs) and the leaf-like organs are elongated.
They then overexpress BOP1 and BOP2. Only weak expressors survived and they were shoots with short blades. Nice result.
[Answer] Thank you!

Is the ligule expression and function truly independent as they state?
[Answer] The *in situ* analysis showed that expression of the *OsBOP*s initiates in two discrete regions, the base of leaf primordia and the position of the incipient boundary region, suggesting that the onset of expression in these regions is independently controlled. However, a better understanding of the mechanisms regulating the expression of the *OsBOP*s is required to give a conclusive answer.
In addition to the position of the incipient boundary organs, the final size of the sheath and blade are strongly affected by subsequent cell divisions and cell elongation. We think it likely that *OsBOP*s are involved in the control of these aspects.

Because of the change in expression levels of the BOPs from embryos to later stages, they follow the effect of miR156, which is expressed at high levels in the first leaf. They make miM156 and mSPL14 plants and see a blade forming in the first leaf, similar to the bop mutant. These results place the SPL14 upstream of bop. They crossed the mSPL14 plant to the 35S:BOP and saw that the BOP was epistatic, again placing BOP downstream.

Minor comments FIg 2 panel B. is it OsBOP2/ or should it be 2/3?
[Answer] We corrected the labels in Fig. 2a and b.

Page 7. Line 129. Do they mean sheath when they say leaf?
[Answer] In this sentence, we indicate leaf primordia at the P3 stage or earlier, when sheath blade differentiation is incomplete. Therefore, we used ‘leaf’ instead of ‘leaf sheath’.

Page 8. Line 135. I think they mean proximal here. Don’t the BOPs suppress blade in the proximal region (where there is sheath)?
[Answer] *OsBOP*s promote sheath differentiation on the proximal side and probably suppress blade differentiation on the proximal side at the same time. In addition, in this paper, we propose that

*OsBOP*s suppress blade differentiation at the distal position based on the finding that an ectopic leaf blade differentiates in the first leaf of the *osbop3* mutant, and in the spikelet organs in *osbop2 osbop3* mutants.

I don't know if the "knots" adds anything in Fig 6h-i. It is hard to make out what we are looking at.
[Answer] We agree with this comment. This part indicates a possible interaction between *OsBOP*s and KNOX. Although interesting, this part describing knot formation strays from the main theme of this work. Thus, we removed this part from the revised manuscript.

Figure 7a. They state leaf position, but isn't it leaf 1, leaf 2, etc? I think of position as something that could occur throughout development.
[Answer] Thank you for this suggestion. We mention leaf 1, and leaf 2, --- and F-2, F-1 and F (Flag leaf) in the revised manuscript.

The upper right corner panels of Figure 6 were not mentioned. They seem quite nice – so please discuss them.
[Answer] This shows that all leaves in the *OsBOP1*ox plant show a characteristic leaf sheath even near the proximal tip. We describe this in more detail in the revised manuscript (line 262-263, page 19).

Reviewer #2 (Remarks to the Author):
1. The title is not attractive. It is already known that BOP genes are involved in leaf proximal-distal patterning in Arabidopsis (for example, Ha et al., Genetics, 2010). What' new here is that the temporal change of BOP levels, probably by miR156-SPL aging module, contributes to the heteroblastic leaf trait (the gradual change in sheath/blade ratio) in rice. Therefore, a new title will be better.

[Answer] According to this suggestion, we changed the title to '*BLADE-ON-PETIOLE* genes control temporal and developmentally regulated changes in the sheath:blade ratio of rice leaves'.

2. The phenotypic analyses were of high quality. For the expression analyses, the qRT-PCR results (Fig. 7e and j) are not informative enough. I would like to see a time-course analyses of BOP transcripts by in situ, from leaf 1 to leaf 4, in both wild-type and miR156ox/spl14 mutant background. These results will help us to know how miR156 precisely regulates the BOP expression. Does miR156 spatially regulates BOP？or just quantitatively?
[Answer] We performed the *in situ* hybridization of *OsBOP1* and *OsBOP2/3* in the *mSPL14* lineas suggested (Supplementary Figure 13). Samples are at same stages as shown in Fig. 4d to for WT. The

data indicate that *OsBOP* signals were reduced in *mRPL14*. In particular, reduction was extreme in 48hr after imbibition, the earliest sateg.

3. I think the Fig 5 is not essential, not closely related to the topic of the manuscript. Authors could move it to the supplemental materials.
[Answer] This Figure shows that the control of the sheath:blade ratio by *OsBOP*s is the same in spikelet organs, which are modified leaves. Therefore, we think that this is relevant to the topic and decided to retain Figure 5 as it stands.

4. The introduction: the summary of the miR156-SPL pathway and juvenile-to-adult phase transition in rice could be expanded since the temporal change of sheath/blade ratio is the highlight of the manuscript. For example, the rice COP1/PPS has been shown to play a role in juvenile-to-adult phase transition in rice (Tanaka, TPC, 2011). Line 60-61: there are no references about the role of miR156 in juvenile-to-adult phase transition. There are some suitable review papers from Scott Poethig, Jia-Wei Wang, or Markus Schmid lab.
[Answer] We modified this part of the introduction as suggested (line 62-66; 69-71, page 4).

In summary, the manuscript is interesting and well written. Most of the experiments were performed at a high standard. The emphasis of the temporal regulation of sheath/blade ratio by miR156-BOP will further strengthen the novelty of the manuscript.
[Answer] Thank you for this comment. We modified the title and text so that the temporal regulation of the sheath/blade ratio by miR156-BOP is emphasized more strongly.

Reviewer #3 (Remarks to the Author):
This manuscript describes a role for rice BLADE-ON-PETIOLE genes in proximal-distal leaf patterning. Grass leaves consist of a proximal sheath and a distal blade separated by boundary organs consisting of an auricle and ligule. CRISPR-Cas9 directed mutations were induced to generate single, double, and triple mutations in OsBOP1, OsBOP2, and OsBOP3. 35S:OsBOP1 transgenic plants were also generated. Analysis of these loss-and gain-of-function mutants showed that BOPs are collectively required for the formation of boundary structures as well as promote sheath cell identity and repress blade development, particularly in the first leaf where BOPs expression levels are highest. The authors provide convincing evidence that this expression pattern is under the influence of miR156 and SPL family genes that control age-dependent changes associated with phase transition in plants. These characterizations of BOP activity align well with roles in eudicot plants and regulation by miR156/SPLs for leaf shaping is new and interesting. This well-written paper sheds light on how monocot leaves are patterned and illustrates the strong potential of CRISPR/Cas9 technology for

reverse genetic studies in crop plants. The weakness is that the mechanism of SPL regulation of BOPs remains unclear and no link is made to domestication traits in rice.

[Answer] We appreciate the recommendation. We agree that the mechanism by which the expression of *OsBOP*s is regulated by SPL is an important aspect that needs to be clarified. We are currently studying this problem.

Minor comments:

1.Abstract, temporal changes in expression of OsBOPs CORRELATE with developmental changes in the sheath:blade ratio. We cannot say they are responsible without additional data. Further, it may be premature to conclude the OsBOPs are the main regulators of proximal-distal patterning rice leaf development since loss-of-function mutations in OsBOPs mainly affect the first leaf.

[Answer] We agree that what we showed is the correlation of the pattern of developmental change of the expression pattern of the *OsBOP*s and the ratio of sheath to the total leaf length. However, we also showed that the proportion of the sheath changes depending on the level of *OsBOP*s expression in a series of loss-of-function mutants and ox plants. Therefore, combining these results, we think our conclusion is plausible. Loss-of-function mutants of *OsBOP*s have effects throughout development. As shown in Fig, the leaves in the triple mutant consist solely of the leaf sheath, although the phenotype is not clear in single and double mutants during the later vegetative stage.

2.A developmental series of leaves for osbop combination mutants showing leaf shape changes should be included (to compare to Supplemental Figure 1 which shows pattern for wild-type leaves). Are blades in the double and triple osbop mutants wider than in WT? There do not appear to be any significant changes in the sheath length according to Figure 3. It is difficult to process all these dimensions without a leaf series.

[Answer] We added data showing leaf shape in the single, double and triple mutants from the first to fourth leaf stages (Fig 3i; Supplementary Fig. 7). The width of the leaf blade is not significantly affected in the mutants.

3.Supplementary Figure 2. The phylogenetic tree is constructed using a neighbor joining method, which is considered less reliable compared to bayesian/maximum likelihood trees for inferring evolutionary histories. The tree should be redrawn. Related to that, the authors do not explain the significance of a grass-specific clade for BOPs. Is this clade predicted to have a specific function in monocots that sets them apart from eudicot BOPs?

[Answer] We redrew the phylogenetic tree. The *OsBOP2* and *OsBOP3* genes in the monocot-specific clade are expressed in the spikelet but *OsBOP1* is not. Thus, one possibility is that the monocot-specific *BOP*s may be involved in the control of spikelet development.

4.The authors propose that OsSPLs repress OsBOP expression allowing blades to develop in leaf 2 and higher (model/text). How then do we account for rice spl8/liguleless1 mutants that lack boundary structures similar to osbop loss-of-function mutants?

[Answer] In the *spl8/liguleless1* mutants of rice, the ligules and auricles are absent, however, the blades and sheaths differentiate normally. We have not studied the interaction between *SPL18* and *OsBOP*s. A plausible possibility is that *SPL18* and *OsBOP*s independently control development of the boundary organs, or that *SPL18* works downstream of *OsBOP*s in boundary organ differentiation. *SPL18* does not contain the target site of miR156, again suggesting that *SPL18* is not in the miR156-SPL-BOP pathway we found in this study.

5.The in situ pictures do not make it clear if OsBOP expression is enriched in the sheath relative to the blade on individual leaves. Any evidence that KNOX genes are ectopically expressed in the sheath region of rice leaves as shown previously for Atbop1 bop2 mutants?

[Answer] We showed that *OsBOP*s are expressed in the whole area of the leaf primordia in the leaves formed during the early vegetative stage. However, *OsBOP* expression is restricted in the sheath region during the adult vegetative stage, as shown in Fig 2. We performed RNAseq analysis, however, no significant changes in *KNOX* expression were detected (not shown). As we mentioned in the Discussion, although the phenotype was initially interpreted as ectopic meristematic ability in the proximal region of leaves, recent studies suggest that proximal-distal patterning is affected in Arabidopsis bop mutants (reference 38, Ichihashi et al., 2011).

6.Supplemental Figure 1. Age of plants is not indicated. Yellow arrowheads and number labels are missing on this figure.

[Answer] The structure of the leaves was measured independently when each leaf reached its final size. We added this explanation to the figure legend. We added the yellow arrowheads and number labels.

7.Figure 3. Arrow color indicating the ectopic leaf that forms on the sheath should be clearly identified in the Figure Legend. Figure 3b. The bar graph is missing the triple mutant. Figure 3n. Square of close-up region should be black (not yellow).

[Answer] We did as you suggested regarding the ectopic leaf. In addition, we added the triple mutant data and corrected the color of the square.

8.Line 188: These data imply….

[Answer] We corrected this.

9.Figure 4bc, Figure 7, Supplementary Figure 10. Show significant differences on the graphs with asterisks.

[Answer] We marked the significant differences as suggested.

10.Figure 2. The labels for OsBOP1 in (a) and OsBOP2/3 in (b) have slipped off the panels. Also, panel labels for e,f,g are outside the panels and whereas the rest of the letters are inside.

[Answer] We corrected the labels in a and b. Regarding e, f, and g, each contains three photos, therefore the labels are outside the photo. To avoid confusion, we modified figures e to g.

11.Supplementary Figure 8. Scale bar for osbop2-1 is slightly thinner than the rest.

[Answer] We corrected this.

12.Supplementary Figure 3. Are the guide RNA target sites located in the BTB/POZ domain? Indicate.

[Answer] We indicated the BTB/POZ domain in Supplementary Figure S3a.

13.Figure 5d. Why is the OsBOP2/3 signal present as punctate dots?

[Answer] These dots are primordia of spikelet organs, such as glumes, rudimentary glumes, the lemma and the palea. Because of their small size during development, they look like dots in the longitudinal sections.

14.BOP knockouts are expected to exhibit defects in flower patterning and abscission. The authors do not specify.

[Answer] The triple *bop* mutant does not form an inflorescence. Therefore, neither flower patterning and abscission zone nor seed dispersal can be observed. Inflorescences in the single and double mutants are smaller than in wt, however, we did not observe clear defects in flower patterning. We added this description to the text (line 143-145, page 8).

15.Materials and Methods. Plant materials. Growth conditions for the rice plants are not given. Include a greater description of how the double and triple mutants were generated after CRISPR/Cas9 singles were found. How many plants were screened to find the initial mutations? Real-time PCR. Include a greater description of conditions used for qPCR, primer design and validation methods, specific statements about the number of biological replicates and normalization methods. Supplementary Table 1. Add unique identifier numbers for rice genes if possible.

[Answer] We added this information in Methods section.

REVIEWERS' COMMENTS:

Reviewer #1 (Remarks to the Author):

I was pretty happy with the original manuscript and am pleased with the changes they have made. The work is stellar and publication of this work will impact others in the field. Sarah Hake

Reviewer #2 (Remarks to the Author):

I am satisfied with authors' response. I think the paper could be accepted at current form.

Reviewer #3 (Remarks to the Author):

I am satisfied with the changes, just a few minor comments remaining.

Line 286. In contrast, expression of non-target OsSPL genes including SPL8 was not significantly changed. [This is actually an interesting finding, that ligule and oracle identity control might be under separate SPL/BOP control].

Line 492. Summary
Provide the unique gene identifiers for OsBOPs e.g.OsBOP1 = Os01g0948900, OsBOP2 = 0s11g0141900, OsBOP3= Os12g0138500 in Materials and Methods. Add the unique identifier for OsUBQ into Supplementary Table S2. No locus info turned up when I searched the Rice Annotation Project Database for OsBOPs or OsUBQ.

Responses to REVIEWERS' COMMENTS:

Reviewer #1 (Remarks to the Author):

I was pretty happy with the original manuscript and am pleased with the changes they have made. The work is stellar and publication of this work will impact others in the field. Sarah Hake

Reviewer #2 (Remarks to the Author):

I am satisfied with authors' response. I think the paper could be accepted at current form.

Reviewer #3 (Remarks to the Author):

I am satisfied with the changes, just a few minor comments remaining.

Line 286. In contrast, expression of non-target OsSPL genes including SPL8 was not significantly changed. [This is actually an interesting finding, that ligule and oracle identity control might be under separate SPL/BOP control].

[Response] Thank you. We are analyzing this in more detail.

Line 492. Summary

Provide the unique gene identifiers for OsBOPs e.g.OsBOP1 = Os01g0948900, OsBOP2 = 0s11g0141900, OsBOP3= Os12g0138500 in Materials and Methods. Add the unique identifier for OsUBQ into Supplementary Table S2. No locus info turned up when I searched the Rice Annotation Project Database for OsBOPs or OsUBQ.

[Response] We added these information in Supplementary Table 2.
